# Imaging the choroidal microvasculature in intensive and high dependency care unit patients: a pilot study

**DOI:** 10.1136/bmjopen-2025-109656

**Published:** 2026-02-25

**Authors:** George Max Cooper, Jamie Burke, Charlene Hamid, Emily Godden, Neeraj Dhaun, Stuart King, Tom MacGillivray, Kenneth Baillie, David M Griffith, Ian J C MacCormick

**Affiliations:** 1 The University of Edinburgh Medical School, Edinburgh, Scotland, UK; 2 School of Mathematics and Maxwell Institute for Mathematical Sciences, The University of Edinburgh School of Mathematics, Edinburgh, Scotland, UK; 3 Robert O Curle Ophthalmology Suite, The University of Edinburgh Centre for Inflammation Research, Edinburgh, Scotland, UK; 4 Clinical Research Imaging Centre, The University of Edinburgh Centre for Clinical Brain Sciences, Edinburgh, Scotland, UK; 5 British Heart Foundation Centre for Cardiovascular Science, The University of Edinburgh Deanery of Clinical Sciences, Edinburgh, Scotland, UK; 6 Edinburgh Clinical Research Facility, Edinburgh, Scotland, UK; 7 Baillie Gifford Pandemic Science Hub, The University of Edinburgh The Roslin Institute, Edinburgh, Scotland, UK; 8 Anaesthesia, Critical Care and Pain, Royal Infirmary of Edinburgh, Edinburgh, Scotland, UK; 9 Department of Anaesthesia, Critical Care and Pain, Molecular, Genetics and Population Health Sciences, The University of Edinburgh School of Molecular and Clinical Medicine, Edinburgh, Scotland, UK; 10 The University of Edinburgh Centre for Clinical Brain Sciences, Edinburgh, Scotland, UK; 11 The University of Edinburgh Institute for Adaptive and Neural Computation, Edinburgh, Scotland, UK

**Keywords:** INTENSIVE & CRITICAL CARE, OPHTHALMOLOGY, Shock, Septic, Physiology

## Abstract

**Abstract:**

**Objectives:**

Microcirculatory dysfunction drives the end-organ pathophysiology of circulatory shock but is not reflected within existing clinical indices of perfusion, such as blood pressure. The choroidal vasculature of the retina can be measured non-invasively and we hypothesised that this may reflect dysfunction in other organs. We tested the feasibility of measuring the choroid in intensive care and explored associations between choroidal measurements and clinical parameters.

**Design:**

A pilot study of optical coherence tomography conducted in a sample of general intensive care unit (ICU) patients.

**Setting:**

A tertiary mixed ICU within the UK.

**Participants:**

15 patients were recruited. One patient was excluded following withdrawal of active treatment. 12/14 (86%) of the remaining patients had successful baseline imaging and 6 (40%) of these had follow-up imaging within intensive care. These patients had a mean age of 56.3 years, were 71% (10/14) male and mean Acute Physiology and Chronic Health Evaluation 2 (APACHE2) score on ICU admission was 20.4.

**Outcome measures:**

Choroidal anatomy, including choroidal and suprachoroidal thickness, as well as volumetric analysis of intrachoroidal blood vessels, was assessed using automated image segmentation along with clinical, physiological and biochemical data at ICU admission and after an interval of 12–72 hours. Feasibility and safety data were assessed throughout ICU admission.

**Results:**

Baseline choroidal vascular index and choroidal thickness were positively associated with fluid balance, and negatively with APACHE2 score, haematocrit and albumin content. A measurable suprachoroidal space was seen in nine (75%) patients (range 25.0–110.0 microns) and was inversely associated with heart rate. There was substantial intraindividual variation in choroidal measurements over time. There were no safety concerns.

**Conclusions:**

Measuring the choroid is feasible in patients with Intensive Care Society Level 2 or Level 3 requirements. The suprachoroidal space may be markedly enlarged in these patients. Exploratory associations with systemic variables suggest that the choroid may provide information about the microvascular function of other major organs. Size and change of choroidal measurements may reflect perfusion pressure and vascular leakage.

**STRENGTHS AND LIMITATIONS OF THIS STUDY**

- We report feasibility of optical coherence tomography (OCT) in a group of intensive care unit (ICU) patients and suggest possible associations with markers of systemic disease.
- We measured the choroid from OCT images with a fully-automated, open-source analysis package to provide robust anatomical data.
- Our sample included a wide range of diagnoses, so our results are not specific to any particular disease.
- Our sample size was limited, analyses were hypothesis-generating and our findings need to be reproduced in further studies that adjust for timing of image acquisition, refractive error and severity of illness.

## Introduction

### Background

 Circulatory shock is a life-threatening failure of central organ perfusion and affects an estimated 30% of patients in intensive care.^1^ Current management aims to improve perfusion by optimising cardiac output, peripheral vascular resistance and circulating volume. These are effective at restoring large vessel physiological measurements, such as blood pressure. However, early goal-directed therapies to restore large vessel parameters appear to be ineffective^2^ or harmful, perhaps because large vessel metrics do not necessarily reflect microcirculatory perfusion.^3^ Microvascular function within central organs is directly relevant to perfusion,^4^ and microvascular perfusion of sublingual capillaries appears to be a better predictor of multiorgan failure in haemorrhagic shock than serum lactate or systolic blood pressure.^5^ However, it is difficult to measure the microvascular perfusion of central organs consistently and repeatedly in humans.^6^ The retina is an exception, since it exposes the central nervous system microvasculature to direct, non-invasive optical imaging and retinal anatomy provides consistent landmarks for precise repeated measurements.^7^

The retina is supplied by two vascular networks. The inner retinal circulation perfuses the superficial two-thirds of the retina and is directly visible on ophthalmoscopy. It has a similar blood–tissue barrier, blood flow and oxygen extraction to the brain.^8^ The outer retina is perfused indirectly by the choroid and choriocapillaris across the outer blood–retina barrier (the retinal pigment epithelium). In contrast to the inner retinal and cerebral vasculature, endothelial cells of the choriocapillaris are fenestrated and choroidal blood flow is 10 times that of the brain. The choroidal circulation has parallels with that of the renal cortex.^9^ The choroid can be imaged using commercially available optical coherence tomography (OCT) by using enhanced-depth imaging (EDI) to penetrate the optically dense retinal pigment epithelium.

### Objectives

OCT and OCT angiography (OCTA) have previously been used to image the retina in intensive care unit (ICU) patients^7 10^ but to our knowledge, there are no published data describing the choroid in this group. Choroidal anatomy adapts dynamically to systemic physiology^11^ and may reflect the perfusion of other central organs.^9^ We sought to test the feasibility of measuring the choroid with EDI-OCT in critically ill patients at ICU admission. In this pilot study, we hypothesised that choroidal indices and relevant clinical markers would be perturbed in critically ill patients. We anticipate that addressing this question could help guide further research evaluating retinal biomarkers of central microcirculatory dysfunction in patients with shock.

## Methods

### Design

We consented and recruited patients to the Direct Retinal Imaging for Shock Resuscitation in Critical Ill Adults II study between March 2023 and July 2023. Patients admitted to the ICU and high-dependency units (HDUs) at the Royal Infirmary of Edinburgh, UK, were consecutively screened for eligibility. We followed STROBE (Strengthening the Reporting of Observational Studies in Epidemiology) guidelines.^12^ The study protocol (online supplemental file 1) and participant materials were developed with patient–public input. Given funding and material availability, 15 participants were sought to assess feasibility of choroidal imaging. Broad eligibility criteria were used to facilitate our assessment of feasibility and make our results generalisable to the UK critical care population, rather than any one particular disease.

### Patient and public involvement:

The study protocol (online supplemental file 1) and participant information sheets, and consent forms were developed in collaboration with the Edinburgh Critical Care Patient and Public Involvement (PPI) Group. These involved iterative structured and semistructured discussions with volunteer former patients, carers and relatives, to ensure a feasible and acceptable research burden for the study population. The PPI group was consulted prior to and throughout the Research and Development and National Research Ethics approval processes. They were provided with regular updates throughout the study period and an interim report on the study’s findings.

### Patient eligibility

All patients admitted during the recruitment period were screened for eligibility on critical care admission by departmental research nurses, provided OCT equipment and research staff were available. Patients were eligible if they were aged 16 or older and receiving Intensive Care Society Level 2 or 3 care. Patients were excluded if they were moribund, pregnant, had obvious bilateral ocular pathology or facial trauma precluding imaging, had clinical contraindications to pupil dilation (ie, traumatic brain injury) or if research was likely to disrupt their care.

### Image capture and data collection

Baseline EDI-OCT was performed on each participant’s right eye at recruitment. Follow-up imaging was sought 12–72 hours later using the boom-mounted Heidelberg Spectralis Flex (Heidelberg Engineering, Germany) (imaging protocols in online supplemental file 2). A diagram of this device is demonstrated in figure 1 of Liu *et al*.^10^ Images were acquired by a team including a specialist ophthalmic imager and an ophthalmologist.

**Figure 1:**
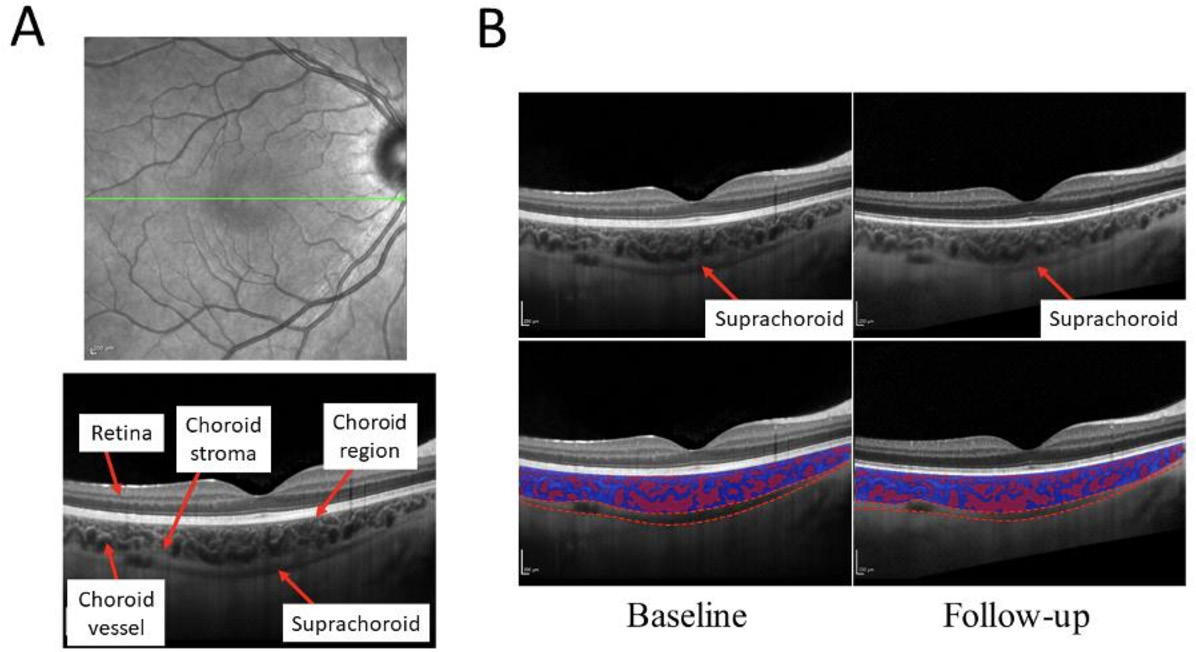
(**A**) En face retinal scan with location of acquisition in green (top), and OCT B-scan with landmarks annotated (bottom). (**B**) Patient with measurable suprachoroid (top), with observed change in suprachoroidal space thickness between time points (bottom). OCT, optical coherence tomography.

Retinal imaging is non-invasive and generally low risk. However, use within ICU/HDU could potentially interfere with standard care. Similarly, pupil dilation carries a small risk of precipitating acute angle closure and can interfere with neurological observations. Therefore, we performed imaging under the supervision of critical care nursing staff and assessed anterior chamber depth prior to pupil dilatation (tropicamide 1%).^13^ Dilating drops were only applied to the right pupil, preserving reactions within the other eye. Imaging was performed under the supervision of critical care nursing staff with vital signs monitored throughout for deterioration. In each case, clinical staff had the opportunity to raise safety concerns at any point during or after the imaging period. Follow-up imaging was only done within ICU/HDU. Baseline patient characteristics and clinical data were collected prospectively from clinical records (full variable list in online supplemental file 3).

### Image analysis

We measured the choroid within a 4 mm, horizontal line region of interest centred on the fovea (figure 1A, top green line). The choroid is defined as the space between the hyperreflective Bruch’s membrane (anterior) and sclera (posterior) (figure 1A, bottom). The suprachoroid is a potential space between the choroid and the sclera (figure 1B, top). The choroid is a complex vascular mesh, and the ratio of vascular spaces to whole choroidal volume is defined by the Choroidal Vascular Index (CVI) (figure 1B, bottom red: blue). A 2 mm wide subfoveal region of interest was selected to mitigate potential selection bias from image quality. We report the subfoveal choroidal thickness, subfoveal suprachoroidal thickness and the CVI, which were quantified using published open-source automated or semiautomated tools: DeepGPET (choroid),^14^ GPET (suprachoroid)^15^ and MMCQ (choroidal vessel spaces) to mitigate information biases.^16 17^ Details are available in online supplemental file 4. Missing data were excluded from analyses and imputation was not attempted.

### Statistical analysis

We measured feasibility in terms of acquisition time, number of attempts taken, the subjective experience of individuals performing OCT imaging and a validated image quality index (Heidelberg Q-score).^18^ The Q-score represents the signal-to-noise ratio within a scan, whereby>25 dB is considered ‘Excellent’. We reviewed the distributions of choroidal and clinical data graphically and explored possible pairwise associations with choroidal metrics using scatterplots. We fitted linear or non-linear functions according to this visual assessment. Our analyses are exploratory and hypothesis-generating, and we report 95% CIs rather than p values. No prespecified sensitivity analyses were performed. Online supplemental file 5, figure 1 reports post hoc sensitivity analyses comparing age, physiological, choroidal indices and Q-Score.^19^ Further analyses against partial pressure of oxygen in arterial blood (PaO2), lactate, mean arterial pressure and serum creatinine were underpowered and showed no association (online supplemental file 5, figure 2).

**Figure 2:**
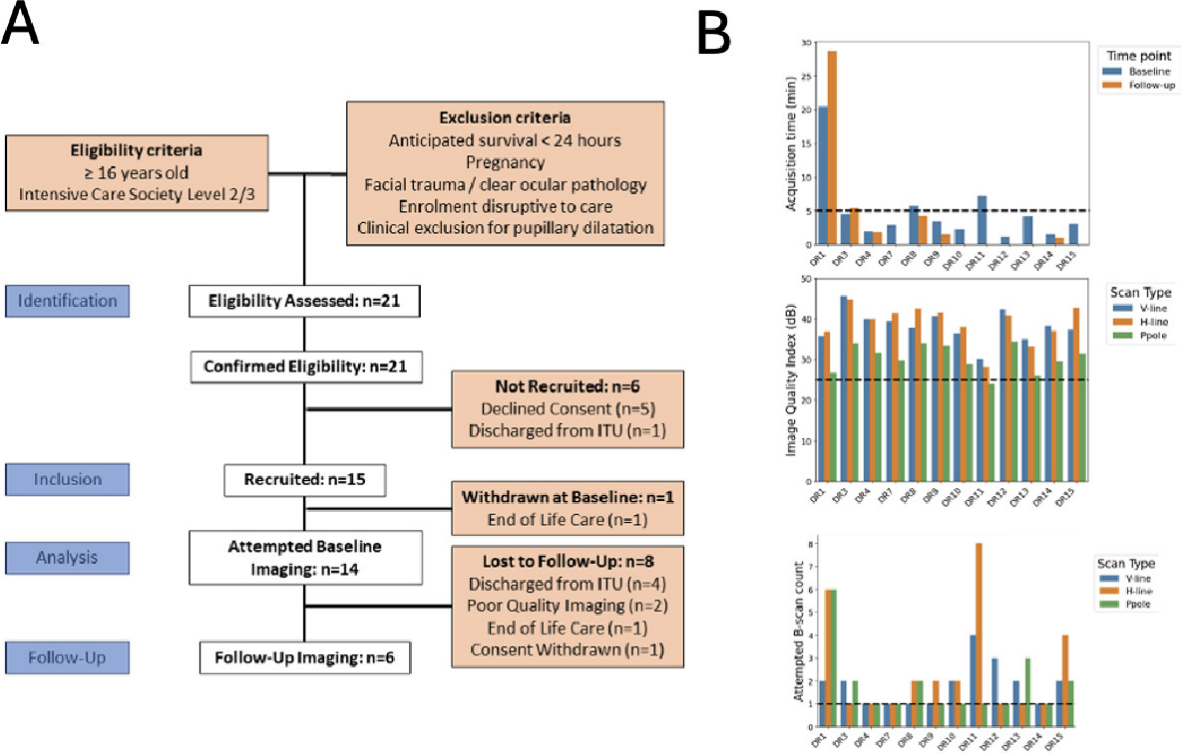
(**A**) Flowchart summarising patient recruitment. (**B**) Per-patient metadata from OCT acquisition describing scan time (top), image quality per scan type (middle) and number of scans attempted per visit (bottom). ITU, intensive therapy unit; OCT, optical coherence tomography.

## Results

### Participants

21 patients were screened and eligible, 15 were recruited, 14 had baseline imaging attempted and 6 also had follow-up imaging (figure 2A). Demographics of participants who had OCT imaging are reported in table 1.

**Table 1:** Patient summary characteristics at baseline

Patient characteristics:	Baseline (n=14)	Follow-up (n=6)	Laboratory reference*
Age (mean, years)	56.2±15.3	47.0±19.0	–
Male sex (frequency, %)	10 (71)	3 (50)	–
Body mass index (mean, kg/m^2^)	28.4±5.9	27.3±7.3	–
APACHE2† Score	20.4±5.9	19±6.6	–
Days since critical care admission (mean)‡	9.1±8.8	11.7±11.0	–
Level 3 care‡	14 (100)	6 (100)	–
Diagnosis (frequency, %)			
Cardiac arrest	1 (7)	0 (0)	–
Type 1 respiratory failure§	2 (14)	2 (34)	–
Overdose	1 (7)	1 (17)	–
Toxic shock syndrome	1 (7)	1 (17)	–
Acute biliary disease¶	2 (14)	1 (17)	–
Ivor-Lewis oesophagectomy	2 (14)	0 (0)	–
Whipple’s procedure	1 (7)	1 (17)	–
Orthotopic liver transplantation	3 (21)	0 (0)	–
Thoraco-aortic aneurysm repair	1 (7)	0 (0)	–
Heart rate (mean, min^−1^)	87.9±16.9	87.8±18.2	–
Mean arterial pressure (mean, mm Hg)	84.0±15.3	87.6±14.3	–
24-hour fluid balance (mean, mL)	460.4±1931.2	−366.5±560.1	–
Cumulative fluid balance since critical care admission (mean, mL)	5376.8±8461.5	6423±11 896.1	–
Highest lactate 24 hours preceding sampling (mean, mmol/L)	1.54±1.00	0.98±0.30	0.5–1.6
Creatinine (mean, mmol/L)	123.6±83.3	117.2±136.7	50–111
Haemoglobin (mean, g/L)	95.2±20.1	92.7±21.3	115–180
Haematocrit (mean)	0.28±0.06	0.28±0.06	0.36–0.52
Albumin (mean, g/L)	18.3±4.2	17.5±3.8	36–47
Organ support (frequency, %)			
Invasive mechanical ventilation (frequency, %)	4 (29)	0 (0)	–
FiO_2_ (mean, %)	31.0±19.5	23.8±5.6	–
SaO_2_ (mean, %)	96.7±2.2	98.0±1.4	–
Lowest PaO2 24 hours preceding sampling (mean, kPa)	7.85±2.18	7.72±2.36	10.2–13.3
Vasopressors (frequency, %)	3 (21)	1 (17)	–
Renal replacement therapy (frequency, %)	0 (0)	0 (0)	–
IV sedation (frequency, %)**	3 (21)	1 (17)	–
Intra-aortic balloon pump (frequency, %)	0 (0)	0 (0)	
Glasgow Coma Scale preceding sampling (median, IQR):	15, 3	15, 0	–
Time of sampling (mean; hh:mm)	13:51±02:15	11:36±01:21	–

Plus–minus data are SD of continuous variables. hh:mm:24-hour time of sample collection.

* Biochemical reference ranges as indicated by local health board (NHS Lothian).

† Acute Physiology and Chronic Health Evaluation Score 2 (APACHE2).

‡ Intensive Care Society Level 2 (high-dependency) or Level 3 (intensive treatment) care.

§ Type 1 respiratory failure: one case secondary to pneumonia (concomitant bacterial and viral).

¶ Biliary disease: one case of sepsis secondary to cholangitis, one patient with acute kidney injury following cholecystitis.

** Sedation including either intravenous propofol or clonidine infusion.

FiO_2_, fractional inspired oxygen; IV, intravenous; NHS, National Health Service; PaO2, partial pressure of oxygen in arterial blood; SaO_2_, arterial oxygen saturation.

### Feasibility

Figure 2B, table 2 show feasibility data. Baseline imaging was attempted in 14 of the 15 recruited participants. One patient was excluded following transition to end of life care. Follow-up imaging was attempted in six patients. Baseline imaging was successful in 12 of 14 (86%) participants and follow-up imaging was successful in all six attempts (100%). One patient did not require topical mydriasis due to their physiological pupil dilatation. Median (IQR) image acquisition time was 2 min (0 min 48 s to 4 min 18 s) in successful cases (figure 2B, top), not including time taken to manoeuvre the device into position. Average (SD) Q-score for horizontal-line scans was 38.9 (4.6) (figure 2B, middle). Median (IQR) number of scan attempts needed per visit was 1.5 (1–2) (figure 2B, bottom). Details of imaging sessions are in table 2. All successful scans were graded ‘Excellent’. The inability to image two participants was attributed to agitation (n=1) and abnormal intraocular anatomy (n=1, high myopia and geographic atrophy). 60% (12/20) total imaging sessions were conducted with patients in supine position. Mean Glasgow Coma Score throughout these was 13.2. Critical care staff did not raise any concerns about the safety of retinal imaging.

**Table 2:** Feasibility data for OCT in critical care settings

Patient ID*	Captured	GCS†	Laterality	Position	Time (m:s)‡	Operator§	SCS visible	Case-specific notes
Patients imaged at baseline								
1	Yes	4	Right	Supine	20:27	A	Yes	Sedated, unable to fixate, large suprachoroid, image artefacts. Camera position affected by ventilator in situ.
2	No	11	Right	Upright	N/A¶	A, B	No	Unable to fixate, cooperate or follow instructions. Camera position affected by ventilator.
3	Yes	15	Right	Upright	04:32	A	Yes	Required suctioning of airway secretions during protocol. Camera position affected by ventilator.
4	Yes	15	Right	Supine	01:59	B	Yes	
5	No	3	Both	Supine	13:32	A	No	Sedated, required saline eye drops for dry eyes. Difficulty locating macula due to previous ophthalmic conditions (AMD). Camera position affected by ventilator.
7	Yes	15	Right	Supine	02:53	A	Yes	Oxygen face mask.
8	Yes	15	Right	Upright	05:44	A	Yes	Camera position affected by nebuliser in situ. Required suctioning midprotocol. Bedside access blocked ipsilaterally.
9	Yes	15	Right	Upright	03:26	A	Yes	Lack of head support due to upright positioning led to difficulty fixating.
10	Yes	15	Right	Upright	02:19	A	Yes	Patient hearing impaired with difficulty fixating.
11	Yes	10	Right	Supine	07:15	A	No	Required saline eye drops for dry eyes. Partially sedated. Highly myopic eyes, large area of peripapillary atrophy. Difficultly fixating. Camera position affected by ventilator.
12	Yes	15	Right	Upright	01:11	B	No	Poor cooperation and fixation due to patient discomfort.
13	Yes	15	Right	Upright	04:09	A	No	Patient drowsy with difficulty fixating.
14	Yes	15	Right	Supine	01:30	A	Yes	
15	Yes	15	Right	Supine	03:04	A	Yes	Difficulty imaging deep choroid with enhanced depth OCT.
Patients imaged at follow-up								
1	Yes	11	Right	Supine	28:40	A	Yes	Sedated, unable to fixate, Camera position affected by ventilator in situ.
3	Yes	15	Right	Supine	05:27	A	Yes	
4	Yes	15	Right	Supine	01:52	A	Yes	Protocol completed despite OCT power supply issues.
8	Yes	15	Right	Supine	04:14	B	Yes	Horizontal imaging repeated to improve capture quality.
9	Yes	15	Right	Supine	01:32	A	Yes	OCT imaging facilitated by supine position.
14	Yes	15	Right	Upright	01:04	A	Yes	

Notes: all recruitment and imaging took place in ICU. There were no adverse events from imaging, and no case-specific comments imply imaging was completed without issue.

* Patient 6 was excluded before baseline imaging due to clinical deterioration and end of life care.

† Glasgow Coma Scale (0–15).

‡ Time estimated as the difference in acquisition time between first and last scan acquired.

§ Operators A were authors CH, JB and B were authors EG, JB.

¶ Only one scan was collected so timing could not be estimated.

AMD, Age related macular degeneration; ICU, Intensive Therapy Unit (Level 3 care); m:s, minutes:seconds; OCT, optical coherence tomography; SCS, suprachoroidal space.

### Choroidal variation

**Figure 3:**
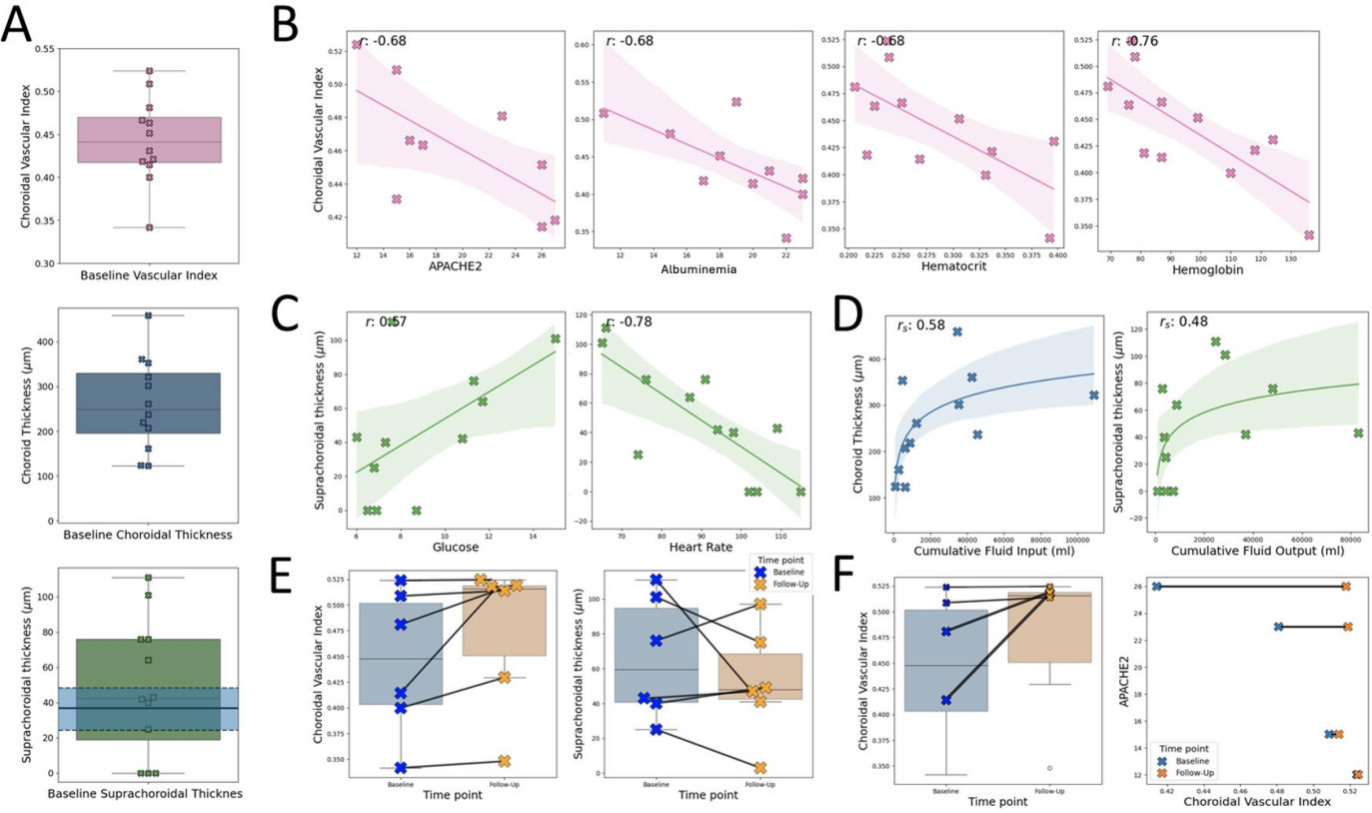
(**A**) Choroidal variation at baseline for CVI (top), subfoveal thickness (middle) and suprachoroidal thickness (bottom), with shaded blue region representing median and IQR from Yiu *et al*,^20^ (**B**) Observed trends between twelve baseline CVI and APACHE2 (n=9), albuminaemia (g/L, n=10), haematocrit (%, n=12) and haemoglobin (g/L, n=12). Missing data were excluded from plots. (**C**) Observed trends between suprachoroidal thickness with glucose (mmol/L, n=11) and heart rate (bpm, n=12). (**D**) Observed, non-linear and monotonic trends between choroid thickness and cumulative fluid input (mL, n=12) and suprachoroidal thickness and cumulative fluid output (mL, n=12). (**E**) Observed change in CVI and suprachoroidal thickness between baseline and follow-up time points (n=6). (**F**) Magnitude of CVI change appeared to increase as a function of APACHE2 score (two patients of the six who were followed up did not have an APACHE2 score). APACHE2, Acute Physiology and Chronic Health Evaluation 2; CVI, Choroidal Vascular Index.

Baseline choroidal thickness, CVI and suprachoroidal space thickness are shown in figure 3A. The suprachoroidal space was visible in 9 (75%) of the 12 participants with successful baseline scans. In some cases, it was markedly enlarged (figure 3B), with the IQR notably larger than previously reported in a healthy cohort^20^ (figure 3A, bottom shaded blue).

Within these 12 patients, baseline CVI decreased with increasing Acute Physiology and Chronic Health Evaluation (APACHE) 2 score (r= –0.68, not calculated in three patients), albuminaemia (r=−0.68, not measured in two patients), haematocrit (r= –0.68) and haemoglobin (r= –0.76) (figure 3B). Choroidal thickness was inversely associated with haemoglobin (r –0.57). Suprachoroidal thickness appeared to be positively associated with glucose (r= +0.57) and negatively associated with heart rate (r= –0.78) (figure 3C). Baseline choroidal measurements appeared to have positive non-linear, monotonic (demonstrated by Spearman’s) associations with cumulative fluid input and output up to the point of imaging (input fluid: choroidal thickness, s=+0.58; suprachoroidal space thickness, s=+0.47, CVI s=+0.57; output fluid: choroidal thickness, s=+0.58; suprachoroidal space thickness, s=+0.48, CVI s=+0.55). Some notable examples are shown in figure 3D.

In the six participants with follow-up imaging, CVI and suprachoroidal thickness changed substantially in some individuals (figure 3E). The largest increases in CVI appeared to be in patients with the highest APACHE2 scores (figure 3F). There were no obvious associations with age or systemic blood pressure. A full list of pairwise comparisons with Pearson/Spearman correlations is shown in online supplemental file 6.

## Discussion

### Key results

We found that EDI-OCT imaging of the choroid is feasible in ICU/HDU patients and reveals substantial variation in the size and vascularity (CVI) of the choroid in this cohort. Our exploratory results suggest that choroidal measurements may reflect systemic fluid status and rheology, and that EDI-OCT is sensitive to changes within individuals over time.

We also found that the suprachoroidal space can be markedly enlarged in this patient group. This frequency (75%) and magnitude of suprachoroidal space is unusual compared with the general population. A thin suprachoroidal space may occur in up to 44% of healthy people aged 55–85 years.^20^ However, an obviously visible space is usually associated with ocular disease such as severely low eye pressure or retinochoroidal inflammation.^21^

### Limitations and generalisability

The primary limitations of this pilot study are its sample size (n=14) and loss to follow-up (57%). In addition, we did not account for the influence of refractive error, or time of day, on measurements of the choroid.^22 23^ However, despite these, we demonstrate the feasibility of EDI-OCT within a highly generalisable population of critically unwell patients. Although only 14 patients were recruited, we successfully imaged 86% (n=12/14) across diverse medical and surgical critical care admissions. Imaging with Spectralis Flex is feasible but requires two or three operators and the cooperation of the bedside multidisciplinary team. Reflecting this constraint and the pragmatic limitations of this study, the mean time from critical care admission to imaging was 9.1 days, which limited the utility and applicability of certain biomarkers (eg, lactate, vasopressor requirement) following the initial stabilisation of these patients. Future work should aim to image patients closer to or during admission, where the effects of acute physiological disturbances may be more apparent, while also accounting for any variation from axial eye length and refractive error, and time of day, all of which can influence choroidal thickness.^22 23^

We anticipate that the size, cost, imaging depth and ease of use of portable and handheld OCT equipment is likely to improve over time.^24^ The advent and improvement of handheld OCT devices could facilitate diagnostic ergonomics, helping create a potential role for ocular microcirculatory biomarkers in the management of critically ill patients, throughout both their inpatient admission and follow-up.^25 26^ Open-source analytics for retinochoroidal OCT images would further facilitate this, to create a ‘closed-loop’ device for non-expert users.^14^,^16^ The primary source of attrition was discharge from critical care (50%, n=4/8) and, in addition to incorporating handheld OCT where feasible, future work should include administrative and ethical provisions to follow patients in lower level inpatient care settings.^27^

Despite these limitations, there were several methodological strengths to this work. First, to the best of our knowledge, this is the first reported characterisation of the choroidal vasculature in critical care patients. In quantifying the choroidal space, a robust and reproducible analytical methodology was used, facilitating future work. Involvement of patient–public partnership methodologies when developing the study protocol and participant materials served to mitigate sampling biases and facilitate recruitment. Finally, while the high degree of heterogeneity limited the interpretation and applicability of our choroidal evaluation, this study demonstrated a high degree of generalisability to UK critical care settings, supporting the future evaluation of EDI-OCT across the spectrum of critical illness in multicentre settings.^28^ Future work will seek to evaluate intraocular structures within more homogenous patient populations.

### Interpretations

To our knowledge, this is the first study to report choroidal imaging in this setting, and across multiple diagnoses. A few studies have reported retinal imaging in ICU,^7 10^ or after discharge from ICU.^29 30^ Courtie *et al*^7^ found changes in retinal blood flow using OCTA in patients undergoing elective oesophagectomy, supporting our findings of abnormal intraocular anatomy in critical illness. Additionally, Liu *et al*^10^ demonstrated OCT and OCTA feasibility in a similar setting. However, due to prolonged acquisition time, OCTA imaging could only be performed in 56% of patients. Our study extends from those results by investigating a larger cohort and suggesting the feasibility of EDI-OCT of the choroid, and we found that imaging the choroid with EDI-OCT was possible in 86% (12/14) of participants. Notably, all our successful scans had an ‘Excellent’ Q-Score, signifying a robust and feasible protocol for future characterisation of the choroid within disease-specific populations.

Measurements of the retina and choroid may help evaluate microcirculatory dysfunction by providing information about small vessels that is not available from traditional macrovascular and systemic markers of perfusion. These have limited applicability to the microvascular circulatory system—for example, aggressive fluid resuscitation through an early goal directed therapy approach did not influence mortality and increased healthcare costs, and in subsets of patients it may cause harm.^2 3 31^ Effective assessment of microcirculatory function may help clinicians understand and ultimately manage shock better, but current techniques to assess the microcirculation are either overly invasive or do not reflect the physiology of vital organs.^6^ Compared with sublingual sidestream-dark field microscopy,^6^ OCT has the advantage of precise spatial registration of images across time and is therefore able to detect micron-scale changes. However, the interpretation of choroidal measurements must also be balanced against both the diverse physiological challenges of ICU admission (including organ dysfunction), the possibility of variation in subfoveal choroidal thickness with time of day and with refractive error.^23^ Future studies would be strengthened by taking time of choroidal measurements into account, as well as refractive error. Although we have focused on using EDI-OCT to measure the choroid, it is possible to assess choroidal vascularity with OCTA.^32^ If patients are able to comply with the longer acquisition time of OCTA compared with OCT, this may provide information that is superior or complementary to OCT.

## Conclusions

This research demonstrates that high quality EDI-OCT can be performed on patients admitted to intensive care settings, without compromising patient care. We report hypothesis-generating findings that identify possible choroidal microcirculatory abnormalities across a highly generalisable population of critically unwell patients. Future work will seek to better characterise the choroid within disease-specific critical care populations (eg, septic shock). It is hoped that improved understanding of vital organ microcirculation will provide new therapeutic paradigms for critically unwell patients.

## Supplementary material

10.1136/bmjopen-2025-109656 online supplemental file 1

10.1136/bmjopen-2025-109656 online supplemental file 2

10.1136/bmjopen-2025-109656 online supplemental file 3

10.1136/bmjopen-2025-109656 online supplemental file 4

10.1136/bmjopen-2025-109656 online supplemental file 5

10.1136/bmjopen-2025-109656 online supplemental file 6

## Data Availability

Data are available upon reasonable request.
