# Supplementary material for: Imaging the choroidal microvasculature in intensive and high dependency care unit patients: a pilot study
Source: BMJ Open. 2026 Feb 25;16(2):e109656. doi: 10.1136/bmjopen-2025-109656 (PMC12958972; doi:10.1136/bmjopen-2025-109656)
Supplement: online supplemental file 1 [file bmjopen-16-2-s001.pdf]

Non-CTIMP Study Protocol

Direct Retinal Imaging for Shock Resuscitation in Critical Ill Adults- II (D-RISC-ii)

	The University of Edinburgh and Lothian Health Board<br>ACCORD<br>The Queen's Medical Research Institute<br>47 Little France Crescent<br>Edinburgh<br>EH16 4TJ
Protocol authors	**George Cooper, Dr David Griffith, Dr Ian MacCormick, Dr Tom MacGillivray, and Prof Kenneth Baillie**
Chief Investigator	**Dr David Griffith**
Sponsor number	AC**22073**
REC Number	**22/SS/0055**
Version Number and Date	**Version 2 6/10/2022**

CONTENTS

1 INTRODUCTION 8
1.1 BACKGROUND 8
1.2 RATIONALE FOR STUDY 8
2 STUDY OBJECTIVES 9
2.1 OBJECTIVES 9
2.1.1 Primary Objective 9
2.1.2 Secondary Objectives 9
2.2 ENDPOINTS 9
2.2.1 Primary Endpoint 9
2.2.2 Secondary Endpoints 9
3 STUDY DESIGN 9
4 STUDY POPULATION 9
4.1 NUMBER OF PARTICIPANTS 9
4.2 INCLUSION CRITERIA 9
4.3 EXCLUSION CRITERIA 9
5 PARTICIPANT SELECTION AND ENROLMENT 10
5.1 IDENTIFYING PARTICIPANTS 10
5.2 CONSENTING PARTICIPANTS 10
5.2.1 Withdrawal of study participants 10
5.3 Co-enrolment 10
6 STUDY ASSESSMENTS 10
6.1 STUDY ASSESSMENTS 10
6.2 LONG TERM FOLLOW UP ASSESSMENTS 11
6.3 STORAGE AND ANALYSIS OF SAMPLES 11
7 DATA COLLECTION 11
7.1 Source Data Documentation 11
7.2 Case Report Forms 11
8 STATISTICS AND DATA ANALYSIS 11
8.1 SAMPLE SIZE CALCULATION 11
8.2 PROPOSED ANALYSES 11
9 ADVERSE EVENTS 13
10 OVERSIGHT ARRANGEMENTS 12
10.1 INSPECTION OF RECORDS 12
10.2 RISK ASSESSMENT 12
10.3 STUDY MONITORING AND AUDIT 12
11 GOOD CLINICAL PRACTICE 13
11.1 ETHICAL CONDUCT 13
11.2 INVESTIGATOR RESPONSIBILITIES 13
11.2.1 Informed Consent 13

11.2.2 Study Site Staff 13
11.2.3 Data Recording 13
11.2.4 Investigator Documentation 13
11.2.5 GCP Training 13
11.2.6 Confidentiality 14
11.2.7 Data Protection 14
12 STUDY CONDUCT RESPONSIBILITIES 14
12.1 PROTOCOL AMENDMENTS 14
12.2 MANAGEMENT OF PROTOCOL NON COMPLIANCE 14
12.3 SERIOUS BREACH REQUIREMENTS 15
12.4 STUDY RECORD RETENTION 15
12.5 END OF STUDY 15
12.6 CONTINUATION OF TREATMENT FOLLOWING THE END OF STUDY 15
12.7 INSURANCE AND INDEMNITY 15
13 REPORTING, PUBLICATIONS AND NOTIFICATION OF RESULTS 16
13.1 AUTHORSHIP POLICY 16
14 REFERENCES 16

LIST OF ABBREVIATIONS

**ACCORD**	Academic and Clinical Central Office for Research & Development - Joint office for The University of Edinburgh and Lothian Health Board
**CI**	Chief Investigator
**CRF**	Case Report Form
**GCP**	Good Clinical Practice
**ICH**	International Conference on Harmonisation
**PI**	Principal Investigator
**QA**	Quality Assurance
**REC**	Research Ethics Committee
**SOP**	Standard Operating Procedure
**OCT**	Optical Coherence Tomography

1 INTRODUCTION

1.1 BACKGROUND

Circulatory shock is a non-specific pathophysiological state constituting systemic tissue hypoperfusion. Aetiological subgroups of circulatory shock states can be considered. Broadly, these include: *(1)* hypovolaemic shock: hypoperfusion following haemorrhagic or non-haemorrhagic (e.g., third-space fluid loss) intravascular depletion; *(2)* cardiogenic shock: secondary to insufficient cardiac output (e.g., mechanical, cardiomyopathic or arrhythmogenic pathologies); *(3)* obstructive shock: following extra-cardiac pump failure, commonly right-sided following massive pulmonary embolism; and *(4)* distributive shock: loss of peripheral vascular resistance. Distributive shock can follow diverse pathologies, commonly these include sepsis, severe inflammatory response syndrome (SIRS), neurogenic, anaphylactic, and toxic pathologies. A fifth aetiology of circulatory shock, endocrine, occurs in patients suffering Addisonian or thyrotoxic crises. However, the mechanisms of this have not been fully elucidated and there is substantive pathophysiological overlap with the aforementioned aetiologies.

Holistically, circulatory shock represents a large proportion of morbidity and mortality in acute care settings. In 198 European intensive care units (ICUs, n=3147 patients) Sakr et al. estimated that 1 in 3 patients experienced some type of circulatory shock throughout the course of their ICU admission(1).

Indeed, septic shock and resultant multi-organ failure has been a prominent cause of COVID-19 mortality within ICU settings(2). Although the global burden of circulatory shock in this setting is unlikely to be fully elucidated, in Wuhan approximately 35% of patients of all COVID-19 patients between December 2019 and 26th January 2020 received vasopressor support(3). Furthermore, 95% of patients receiving invasive mechanical variation in New York, between the 3rd and 27th of March 2020 received similar vasoconstrictive support(4).

Current management of circulatory shock is targeted at improving vascular perfusion. Other than in obstructive shock, this involves inotropes and vasopressors to support cardiac output and systematic vascular resistance, with fluid resuscitation indicated in distributive, hypovolaemic and certain endocrine settings (e.g., an Addisonian crisis) to further improve cardiac output through increased intravascular volume, venous return and thus preload.

This treatment philosophy assumes that the microvascular environment is reflective of macrovascular conditions. Microcirculatory dysfunction has been well characterised in both in-vitro and in-vivo settings, the MICROSHOCK study found sublingual perfused vessel density (a measure of microcirculatory function) a better discriminator for multiorgan failure in patients with traumatic haemorrhagic shock: area under curve 0.87 (0.76-0.99) compared with highest recorded lactate 0.69 (0.53-0.84) and cardiac index 0.66 (0.49-0.83)(5). Indeed this dissociation between micro- and macro-circulation may explain the therapeutic failure of early goal-directed therapies (EGDTs), which sought to aggressively pursue macrovascular physiological parameters(6). Although an initial study demonstrated significant reduction in in-hospital mortality, this was not replicated in subsequent validation studies or in individual patient meta-analysis(6–10). The additional 48-hour mortality associated with aggressive fluid resuscitation in African paediatric patients with severe febrile illness highlights how overly aggressive fluid resuscitation may have a deleterious impact on patient outcomes(11).

1.2 RATIONALE FOR STUDY

Recent advancements in optical coherence tomography (OCT) highlighted its potential as a non-invasive, safe, feasible and valid approach to observe and quantify intraocular structures in supine, critically ill, patients(12). The retinal and choroidal vascular beds represent novel microvascular sample sites. Previous microvascular physiological studies in circulatory shock have focussed on sublingual, buccal,

gastric, muscular, and subcutaneous capillary beds however the microvascular circulation is a highly heterogenous environment making interpretation in the clinical setting challenging. (5,13–17).

As part of the physiological response to shock or vasopressor therapy (e.g., norepinephrine) and mediated through the distribution of adrenergic receptors in arterial smooth muscle cells, peripheral and then visceral organ perfusion is sacrificed to preserve central neurological (including ocular structures) and cardiopulmonary function(18). Consequently, we hypothesise that microvascular blood flow in the intra-ocular retinal and choroidal vascular beds will provide a better representation of the vascular environment in vital organs such as the brain, heart and lungs in patients experiencing circulatory shock.

We are trying to develop a hand-held, point of care monitoring device based on optical imaging. As a first step in understanding whether microcirculatory changes in the intra-ocular vasculature can be used to monitor shock progression in critically ill adults, we aim to perform OCT imaging of a heterogenous group of intensive care and high dependency unit patients, to ascertain whether variation in retinal and choroidal structure correlates with disease state and physiological status.

Ultimately, it is hoped that better monitoring of patient fluid status will enable patient-level physiological optimisation of fluid resuscitation, preventing excess morbidity due to fluid overload within the critically ill patient population.

2 STUDY OBJECTIVES

2.1 OBJECTIVES

2.1.1 Primary Objective

To observe OCT appearances of the retina and whether they differ according to fluid balance (cumulative or 24-hour), blood pressure, and circulatory shock diagnosis, in a heterogenous cohort of critically ill adult patients.

2.1.2 Secondary Objectives

1. To observe changes in retinal OCT measurements in critically ill adult patients over time (over a 24-hour observation period)
2. To assess the feasibility of and image quality (by blinded assessors) from performing OCT in a critical care environment, using the Heidelberg Spectralis Flex Module.
3. To assess and attempt to model for possible measurement errors in performing these OCT measurements (e.g., variation in X-, Y-, Z-axis alignment of the probe when attempting visualisation).

2.2 ENDPOINTS

2.2.1 Primary Endpoint

Retinal and choroid thickness measured by OCT

2.2.2 Secondary Endpoints

Change in retinal and choroid thickness between first (baseline) and second measurement (12-72 hours later).

3 STUDY DESIGN

D-RISC-ii is an observational study that will run for 12 months within the critical care unit (comprising intensive care and high dependency care beds) at a single institution (Royal Infirmary of Edinburgh).

Initially, the study will function with a case-series approach, identifying patients, visualising their retinal and choroidal beds with optical coherence tomography, collecting exposure data, and assessing the feasibility of further works in this setting. The primary outcome incorporates a cross-sectional element, as we will attempt to characterise patients with to pre-specified exposure features (circulatory shock diagnosis, blood pressure, fluid balance), according to the dimensions of retinal and choroidal structures, at the initial recruitment sampling timepoint. Our secondary outcome follows a cohort approach, seeking to identify variation in these choroidal and retinal parameters according to exposure.

4 STUDY POPULATION

4.1 NUMBER OF PARTICIPANTS

We will seek to recruit patients from intensive care and high dependency treatment units at one site over a one-year period. We do not propose a maximum number of patients over this time period.

INCLUSION CRITERIA

1. At least 16 years old AND
2. Admitted to intensive care (ICU) or high-dependency care units (HDU) AND
3. A requirement for Intensive Care Society Level 2 or 3 care will be eligible to participate(19).

4.2 EXCLUSION CRITERIA

Patients may be excluded for any of the following criteria:

1. Patient refusal
2. Next of kin or power or attorney unavailability or refusal
3. Anticipated survival <24 hours
4. Pregnancy
5. Confirmed or suspected conditions where there is a clinical exclusion for pupillary dilation: e.g., traumatic brain injury, meningitis, cerebrovascular accident, space-occupying lesion (tumour, encephalitis etc.).
6. Ophthalmologic conditions: facial trauma precluding visualization of both retinas.
7. Imaging not feasible (equipment/ researcher unavailable or disruptive to care provision).

4.3 CO-ENROLMENT

As this study is non-interventional, with limited additional burden to participants (beyond standard care of care in intensive care/ high dependency units), participant co-enrolment will be permitted with other non-interventional and interventional studies. However, to mitigate against unforeseen circumstances, co-enrolment will require mutual agreement between CIs and the Sponsor as per the ACCORD co-enrolment policy.

5 PARTICIPANT SELECTION AND ENROLMENT

5.1 IDENTIFYING PARTICIPANTS

Patients requiring treatment in intensive care (ICU) or high dependency (HDU) units will be identified and screened for eligibility by members of the clinical care team (medical student, nurses, research nurses and physicians).

Once eligibility has been confirmed, the patient or their legal representative (under Adults with Incapacity (Scotland) 2000 Act) will be approached and information about the study provide in written and verbal form. The initial approach will be made by a member of the clinical care team (embedded medical student, research nurses and physicians) with the agreement of the treating physician.

5.2 CONSENTING PARTICIPANTS

A. Individuals with capacity:

Members of the research team will periodically be available to answer any queries and clarify understanding. Subsequently, if agreeable, formal written consent will be requested and documented on the patient's notes.

B. Individuals without capacity:

Where potential participants are considered not to have capacity to consent, consent will be sought from their legal representative under the Adults with Incapacity (Scotland) 2000 Act. Members of the research team will periodically be available to answer any queries and clarify understanding. Subsequently, if agreeable, formal written consent will be requested and documented on the patient's notes.

If the representative is not available in-person at any stage, information will be provided and consent sought by telephone by an experienced/ trained member of the clinical care team (research nurse, senior registrar or consultant) and witnessed by another member of the team. Verbal consent will then be documented in the patient's notes and written consent sought at the first convenient opportunity as above. To minimise distress, representatives will only be approached by telephone with the agreement of the treating physician.

C. Participant regains capacity:

Upon regaining capacity, follow-up consent will be obtained as quickly as feasibly possible, as outlined in Part A.

5.2.1 Withdrawal of Study Participants

Participants are free to withdraw from the study at any point or a participant can be withdrawn by the Investigator. If withdrawal occurs, the primary reason for withdrawal will be

documented in the participant's case report form, if possible. The participant will have the option of withdrawal from:

(i) all aspects of the trial but continued use of data collected up to that point. To safeguard rights, the minimum personally identifiable information possible will be collected.

6 STUDY ASSESSMENTS

6.1 STUDY ASSESSMENTS

Assessment	Screening	Baseline Visualisation	Repeat Visualisation (after 12-72 hours)
Assessment of Eligibility Criteria	☒		
Written informed consent	☒		
Contact details	☒		
Demographic data (e.g., age, sex, race)	☒		
Date/time of hospital admission		☒	
ICU/ HDU admission		☒	
Date/time of ICU/HDU admission		☒	
Diagnosis at admission and any renal, cardiovascular, hepatic, pulmonary or ophthalmologic comorbidities.		☒	
Changes in diagnosis/ status		☒	☒
Fluid balance (24-hour and cumulative)		☒	☒
Clinical assessment of fluid balance		☒	☒
OCT		☒	☒
OCT measurements (time, date, eye dilated, angle of bed, prone/ supine)		☒	☒
Adverse events to study protocol		☒	☒
Organ supportive therapies		☒	☒
Most recent BMI		☒	☒
Most recent APACHE II		☒	☒
24-hour mean PaO2, Lactate, FiO2, CRP.		☒	☒
Pre-sample SaO2, RR, HR, systolic BP,		☒	☒

MAP, Temp, GCS.			
Most recent reticulocytes, haemocrit, Hb, WBC, albumin, serum osmolality.		☒	☒

.

7 DATA COLLECTION

Data will be collected by members of the research team including named medical students and research nurses and supervised by the CI and qualified by training, experience, and good clinical practice (GCP) certification.

The OCT operator will receive training to operate the unit. Imaging should take no longer than 15 minutes and is non-invasive.

7.1 Source Data Documentation

Data will come from the following sources:

1. The electronic patient record (EPR)
2. Paper-based patient records (including clinical observation charts)
3. Direct recording of clinical observations from patient monitors
4. Clinical examination
5. Optical Coherence Tomography (OCT) measurements.

7.2 Case Report Forms

Paper-based case report forms will be used to collect data from sources 1-4. These will be stored together in a locked cupboard in a locked research office in the Royal Infirmary of Edinburgh.

Optical coherence tomography visualisations (from source 5) will be retained locally on an encrypted system that accompanies the Heidelberg Spectralis Flex Module.

7.2.1 Personal Data

We will retain patient name, contact details (including address, email address (if possessed) and contact phone number) on the paper-based case-report form. If necessary, we will also retain similar contact details for the participant’s representative under Adults with Incapacity (Scotland) 2000 act for the purposes of contacting them during the research. other measures collected during the study will be non-identifiable. Identifiable data will be linked with an anonymised study database through a unique identification number.

The paper-based case-report form (and the corresponding study index identifier) will be stored by the research team within a secure filing cabinet within a locked room within NHS Lothian onsite at the Royal Infirmary of Edinburgh. Prof Baillie, Dr MacCormick and George

Cooper (medical student) and other authorised study personell will have access; however, this will be controlled by Dr David Griffith (Chief Investigator).

7.2.2 Data Information Flow

Patient identifiable data will be collected when informed consent is obtained at screening and CHI number will be used to identify the participant during data collection and stored on paper-based case report forms.

Clinical data (Sources 1-4) will be collected on paper-based case report forms (including identifiers). These will be stored at the NHS Lothian onsite (locked facilities where access is restricted to within the research team). This data will be stripped of patient identifying information, indexed by study index identifier, and transcribed into an excel spreadsheet on the NHS Lothian server. Subsequently, this anonymous data will be transferred by secure file transfer protocol to a University of Edinburgh server at the Roslin Institute.

OCT visualisations (Source 5) will initially be stored locally on an encrypted system that accompanies the Heidelberg Spectralis Flex Module. These images will be anonymised and indexed by study index identifier. Following connection of the Heidelberge Spectralis Flex Module to a University of Edinbugh server, the anonymised visualisations will be transferred to the retinal image database which is a located on secure server within the Edinburgh Imaging QMRI facility at the University. Analysis of images will be conducted within the Image Analysis Core laboratory in QMRI using the Heidelberg HeyEx software and the outputs will be transferred to a secure server by password protected data sharing using the University's DataSync system for further analysis at the Roslin Institute under the supervision of Professor Baillie.

Patient identifiable data will be destroyed after 3 years. All other study data will be destroyed after 10 years.

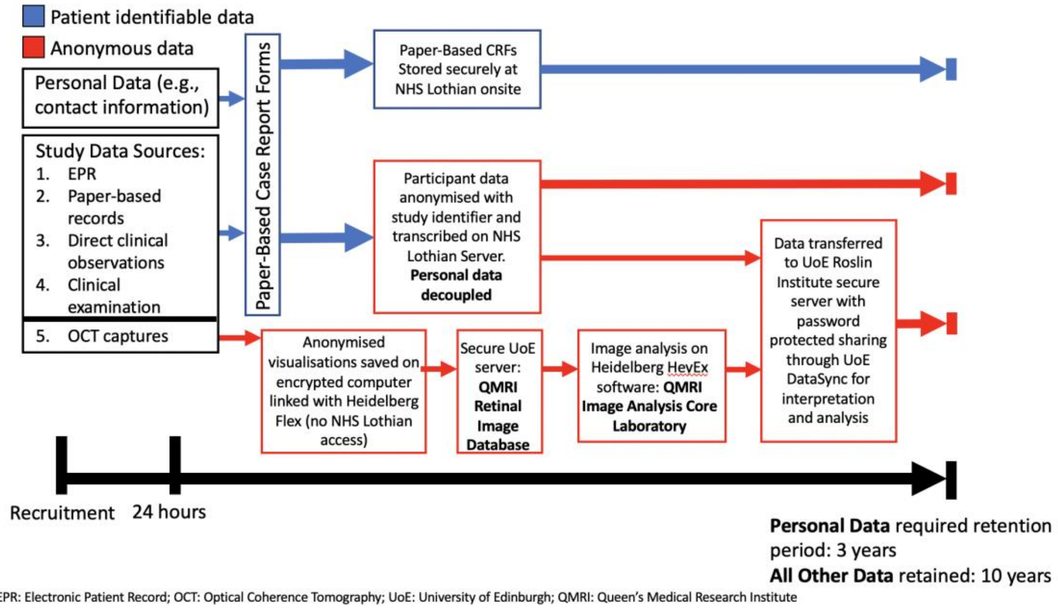

EPR: Electronic Patient Record; OCT: Optical Coherence Tomography; UoE: University of Edinburgh; QMRI: Queen's Medical Research Institute

7.2.3 Transfer of Data

Anonymised visualisations and anonymised participant data may be shared with researchers at other institutions, but any data sharing will be subject to the negotiation of a suitable data-sharing agreement.

7.2.4 Data Controller

The University of Edinburgh and NHS Lothian are joint data controllers along with any other entities involved in delivering the study that may be a data controller in accordance with applicable laws (e.g., the site)

7.2.5 Data Breaches

Any data breaches will be reported to the University of Edinburgh and NHS Lothian Data Protection Officers who will onward report to the relevant authority according to the appropriate timelines if required.

8 STATISTICS AND DATA ANALYSIS

8.1 SAMPLE SIZE CALCULATION

The initial feasibility study (D-RISC) only recruited 2 patients but demonstrated feasibility with some adjustments to the protocol (which have now been implemented, such as less frequent monitoring and a boom operated OCT device).

Consequently, this sample size (at least 15 patients) is one of convenience to allow adequate characterisation of the intra-ocular vasculature of critically ill patients. Once 15 patients have been recruited, we will undertake an interim analysis to consider whether recruitment of additional patients would increase the validity of the dataset.

8.2 PROPOSED ANALYSES

Summary measures to be reported:

As per section 6.1, 35 summary and exposure parameters will be collated throughout the duration of the study.

Method of analysis:

Manual grading of quality of intra-ocular visualisations with OCT will be performed, with unsatisfactory visualisations removed from the dataset.

Quantification of choroidal and retinal thickness will be performed with automated image processing techniques.

Where only an initial set of measurements is captured from a participant, we will not attempt to impute missing values. Instead, we will only analyse that data with respect to other initial captures.

Univariate and multivariate analysis of intra-ocular measurements against clinical data points to ascertain possible differences with respect to our predefined subgroups: patient fluid balance indices (cumulative and 24-hour), blood pressure, and a clinical diagnosis of circulatory shock at initial measurement (according to intention-to-treat principle).

An interim analysis will be performed after data from 15 patients has been recorded to establish whether a larger sample size is necessary to increase the validity of the dataset.

9 ADVERSE EVENTS

Any potential risks and burdens to patients from this study will most likely arise from adverse reactions to the administration of pupil dilatory eye drops (topical mydriasis with tropicamide 1% solution). These are very rare, and attempts will be made to further mitigate against these through the study protocol as outlined below. However, should an adverse event, outcome or reaction come to pass, it will be escalated to the clinical care team as appropriate, documented within patient's clinical notes, the patient's case report form and, with consent and if appropriate a report will be submitted to the Medicines and Healthcare products Regulatory Agency (MHRA) through the British National Formulary (BNF) Yellow Card Scheme.

If this event is perceived to be minor, we will discuss with the patient (including their legal representative, if appropriate) and re-obtain consent to participate. Otherwise, the patient will immediately be withdrawn from the study, and we will seek to obtain consent to retain the participant's data collected up to this point.

1. Adverse events from topical mydriasis will be limited by only applying eye drops to a maximum of one eye per patient.
    - A. Topical mydriasis with tropicamide 1% can rarely cause eye erythema; eye irritation (on prolonged administration); eye pain; headache; hypotension; nausea; syncope.
    - B. Acute closed angle glaucoma. This can occur very rarely following administration of topical mydriasis to dilate pupils. To mitigate this, all patients will be screened for shallow anterior chamber depth using a pen-torch. Such patients will not receive topical mydriasis in this eye on safety grounds.

2. In critically unwell and unconscious patients, pupillary reflexes are used to measure central nervous system (CNS) injury. Dilating the pupil with topical mydriasis prevents this from being measured in the affected eye. Consequently we will take a number of actions to mitigate against the missed diagnosis of CNS injury in participants.
    - A. We will not recruit patients with confirmed or suspected conditions where there is a clinical exclusion for pupillary dilation (e.g. traumatic brain injury, stroke, space occupying lesions (e.g., tumours or encephalitis) or meningitis) or undergoing neurological observation.
    - B. We will ensure topical mydriasis is not applied to one eye and documenting the affected eye, preserving capacity for brainstem monitoring.
    - C. We will use the dilated eye to observe potential signs of neurological injury (e.g., papilloedema), which can be difficult to measure otherwise.

10 OVERSIGHT ARRANGEMENTS

10.1 INSPECTION OF RECORDS

Investigators and institutions involved in the study will permit trial related monitoring and audits on behalf of the sponsor, REC review, and regulatory inspection(s). In the event of audit or monitoring, the Investigator agrees to allow the representatives of the sponsor direct access to all study records and source documentation. In the event of regulatory inspection, the Investigator agrees to allow inspectors direct access to all study records and source documentation.

10.2 STUDY MONITORING AND AUDIT

The ACCORD Sponsor Representative will assess the study to determine if an independent risk assessment is required. If required, the independent risk assessment will be carried out by the ACCORD Quality Assurance Group to determine if an audit should be performed before/during/after the study and, if so, at what frequency.

Risk assessment, if required, will determine if audit by the ACCORD QA group is required. Should audit be required, details will be captured in an audit plan. Audit of Investigator sites, study management activities and study collaborative units, facilities and 3rd parties may be performed.

11 GOOD CLINICAL PRACTICE

11.1 ETHICAL CONDUCT

The study will be conducted in accordance with the principles of the International Conference on Harmonisation Tripartite Guideline for Good Clinical Practice (ICH GCP).

Before the study can commence, all required approvals will be obtained and any conditions of approvals will be met.

11.2 INVESTIGATOR RESPONSIBILITIES

The Investigator is responsible for the overall conduct of the study at the site and compliance with the protocol and any protocol amendments. In accordance with the principles of ICH GCP, the following areas listed in this section are also the responsibility of the Investigator. Responsibilities may be delegated to an appropriate member of study site staff.

11.2.1 Informed Consent

The Investigator is responsible for ensuring informed consent is obtained before any protocol specific procedures are carried out. The decision of a participant to participate in clinical research is voluntary and should be based on a clear understanding of what is involved.

Participants must receive adequate oral and written information – appropriate Participant Information and Informed Consent Forms will be provided. The oral explanation to the participant will be performed by the Investigator or qualified delegated person and must cover all the elements specified in the Participant Information Sheet and Consent Form.

The participant must be given every opportunity to clarify any points they do not understand and, if necessary, ask for more information. The participant must be given sufficient time to

consider the information provided. It should be emphasised that the participant may withdraw their consent to participate at any time without loss of benefits to which they otherwise would be entitled.

The participant will be informed and agree to their medical records being inspected by regulatory authorities and representatives of the sponsor(s).

The Investigator or delegated member of the trial team and the participant will sign and date the Informed Consent Form(s) to confirm that consent has been obtained. The participant will receive a copy of this document and a copy filed in the Investigator Site File (ISF) and participant’s medical notes (if applicable).

11.2.2 Study Site Staff

The Investigator must be familiar with the protocol and the study requirements. It is the Investigator's responsibility to ensure that all staff assisting with the study are adequately informed about the protocol and their trial related duties.

11.2.3 Data Recording

The Principal Investigator is responsible for the quality of the data recorded in the CRF at each Investigator Site.

11.2.4 Investigator Documentation

- The Principal Investigator will ensure that the required documentation is available in local Investigator Site files ISFs.

11.2.5 GCP Training

For non-CTIMP (i.e. non-drug) studies all researchers are encouraged to undertake GCP training in order to understand the principles of GCP. However, this is not a mandatory requirement unless deemed so by the sponsor. GCP training status for all investigators should be indicated in their respective CVs.

11.2.6 Confidentiality

All laboratory specimens, evaluation forms, reports, and other records must be identified in a manner designed to maintain participant confidentiality. All records must be kept in a secure storage area with limited access. Clinical information will not be released without the written permission of the participant. The Investigator and study site staff involved with this study may not disclose or use for any purpose other than performance of the study, any data, record, or other unpublished information, which is confidential or identifiable, and has been disclosed to those individuals for the purpose of the study.. Prior written agreement from the sponsor or its designee must be obtained for the disclosure of any said confidential information to other parties.

11.2.7 Data Protection

All Investigators and study site staff involved with this study must comply with the requirements of the appropriate data protection legislation (including the General Data Protection Regulation and Data Protection Act) with regard to the collection, storage, processing and disclosure of personal information.

Computers used to collate the data will have limited access measures via user names and passwords.

Published results will not contain any personal data and be of a form where individuals are not identified and re-identification is not likely to take place

STUDY CONDUCT RESPONSIBILITIES

11.3 PROTOCOL AMENDMENTS

Any changes in research activity, except those necessary to remove an apparent, immediate hazard to the participant in the case of an urgent safety measure, must be reviewed and approved by the Chief Investigator.

Amendments will be submitted to a sponsor representative for review and authorisation before being submitted in writing to the appropriate REC, and local R&D for approval prior to participants being enrolled into an amended protocol.

11.4 MANAGEMENT OF PROTOCOL NON COMPLIANCE

Prospective protocol deviations, i.e. protocol waivers, will not be approved by the sponsors and therefore will not be implemented, except where necessary to eliminate an immediate hazard to study participants. If this necessitates a subsequent protocol amendment, this should be submitted to the REC, and local R&D for review and approval if appropriate.

Protocol deviations will be recorded in a protocol deviation log and logs will be submitted to the sponsors every 3 months. Each protocol violation will be reported to the sponsor within 3 days of becoming aware of the violation. All protocol deviation logs and violation forms should be emailed to QA@accord.scot

Deviations and violations are non-compliance events discovered after the event has occurred. Deviation logs will be maintained for each site in multi-centre studies. An alternative frequency of deviation log submission to the sponsors may be agreed in writing with the sponsors.

11.5 SERIOUS BREACH REQUIREMENTS

A serious breach is a breach which is likely to effect to a significant degree:

(a) the safety or physical or mental integrity of the participants of the trial; or

(b) the scientific value of the trial.

If a potential serious breach is identified by the Chief investigator, Principal Investigator or delegates, the co-sponsors (seriousbreach@accord.scot) must be notified within 24 hours. It is the responsibility of the co-sponsors to assess the impact of the breach on the scientific value of the trial, to determine whether the incident constitutes a serious breach and report to research ethics committees as necessary.

11.6 STUDY RECORD RETENTION

All study documentation will be kept for a minimum of 3 years from the protocol defined end of study point. When the minimum retention period has elapsed, study documentation will not be destroyed without permission from the sponsor.

11.7 END OF STUDY

The end of study is defined as the last participant's last visit.

The Investigators or the co-sponsor(s) have the right at any time to terminate the study for clinical or administrative reasons.

The end of the study will be reported to the REC, and R+D Office(s) and co-sponsors within 90 days, or 15 days if the study is terminated prematurely. The Investigators will inform participants of the premature study closure and ensure that the appropriate follow up is arranged for all participants involved. End of study notification will be reported to the co-sponsors via email to resgov@accord.scot

A summary report of the study will be provided to the REC within 1 year of the end of the study.

11.8 CONTINUATION OF TREATMENT FOLLOWING THE END OF STUDY

This is an observational study and thus there will be no treatment to continue.

11.9 INSURANCE AND INDEMNITY

The co-sponsors are responsible for ensuring proper provision has been made for insurance or indemnity to cover their liability and the liability of the Chief Investigator and staff.

The following arrangements are in place to fulfil the co-sponsors' responsibilities:

- The Protocol has been designed by the Chief Investigator and researchers employed by the University and collaborators. The University has insurance in place (which includes no-fault compensation) for negligent harm caused by poor protocol design by the Chief Investigator and researchers employed by the University.
- Sites participating in the study will be liable for clinical negligence and other negligent harm to individuals taking part in the study and covered by the duty of care owed to them by the sites concerned. The co-sponsors require individual sites participating in the study to arrange for their own insurance or indemnity in respect of these liabilities.
- Sites which are part of the United Kingdom's National Health Service will have the benefit of NHS Indemnity.
- Sites out with the United Kingdom will be responsible for arranging their own indemnity or insurance for their participation in the study, as well as for compliance with local law applicable to their participation in the study.

12 REPORTING, PUBLICATIONS AND NOTIFICATION OF RESULTS

12.1 AUTHORSHIP POLICY

Ownership of the data arising from this study resides with the study team.

13 REFERENCES

1. Sakr Y, Reinhart K, Vincent J-L, Sprung CL, Moreno R, Ranieri VM, et al. Does dopamine administration in shock influence outcome? Results of the Sepsis Occurrence in Acutely Ill Patients (SOAP) Study*: Critical Care Medicine. 2006 Mar;34(3):589–97.

2. Elezkurtaj S, Greuel S, Ihlow J, Michaelis EG, Bischoff P, Kunze CA, et al. Causes of death and comorbidities in hospitalized patients with COVID-19. Sci Rep. 2021 Feb 19;11(1):4263.

3. Yang X, Yu Y, Xu J, Shu H, Xia J, Liu H, et al. Clinical course and outcomes of critically ill patients with SARS-CoV-2 pneumonia in Wuhan, China: a single-centered, retrospective, observational study. The Lancet Respiratory Medicine. 2020 May 1;8(5):475–81.

4. Goyal P, Choi JJ, Pinheiro LC, Schenck EJ, Chen R, Jabri A, et al. Clinical Characteristics of Covid-19 in New York City. N Engl J Med. 2020 Jun 11;382(24):2372–4.

5. Hutchings SD, Naumann DN, Hopkins P, Mellis C, Riozzi P, Sartini S, et al. Microcirculatory Impairment Is Associated With Multiple Organ Dysfunction Following Traumatic Hemorrhagic Shock: The MICROSHOCK Study. Critical Care Medicine. 2018 Sep;46(9):e889–96.

6. Early, Goal-Directed Therapy for Septic Shock — A Patient-Level Meta-Analysis. New England Journal of Medicine. 2017 Jun 8;376(23):2223–34.

7. Rivers E, Nguyen B, Havstad S, Ressler J, Muzzin A, Knoblich B, et al. Early goal-directed therapy in the treatment of severe sepsis and septic shock. N Engl J Med. 2001 Nov 8;345(19):1368–77.

8. ProCESS Investigators, Yealy DM, Kellum JA, Huang DT, Barnato AE, Weissfeld LA, et al. A randomized trial of protocol-based care for early septic shock. N Engl J Med. 2014 May 1;370(18):1683–93.

9. ARISE Investigators, ANZICS Clinical Trials Group, Peake SL, Delaney A, Bailey M, Bellomo R, et al. Goal-directed resuscitation for patients with early septic shock. N Engl J Med. 2014 Oct 16;371(16):1496–506.

10. Mouncey PR, Osborn TM, Power GS, Harrison DA, Sadique MZ, Grieve RD, et al. Trial of early, goal-directed resuscitation for septic shock. N Engl J Med. 2015 Apr 2;372(14):1301–11.

11. Maitland K, Kiguli S, Opoka RO, Engoru C, Olupot-Olupot P, Akech SO, et al. Mortality after Fluid Bolus in African Children with Severe Infection. New England Journal of Medicine. 2011 Jun 30;364(26):2483–95.

12. Courtie E, Veenith T, Logan A, Denniston AK, Blanch RJ. Retinal blood flow in critical illness and systemic disease: a review. Ann Intensive Care. 2020 Nov 12;10:152.

13. Sakr Y, Gath V, Oishi J, Klinzing S, Simon T-P, Reinhart K, et al. Characterization of buccal microvascular response in patients with septic shock. Eur J Anaesthesiol. 2010 Apr;27(4):388–94.

14. Temmesfeld-Wollbrück B, Szalay A, Mayer K, Olschewski H, Seeger W, Grimminger F. Abnormalities of gastric mucosal oxygenation in septic shock: partial responsiveness to dopexamine. Am J Respir Crit Care Med. 1998 May;157(5 Pt 1):1586–92.

15. Gomersall CD, Joynt GM, Freebairn RC, Hung V, Buckley TA, Oh TE. Resuscitation of critically ill patients based on the results of gastric tonometry: a prospective, randomized, controlled trial. Crit Care Med. 2000 Mar;28(3):607–14.

16. Ikossi DG, Knudson MM, Morabito DJ, Cohen MJ, Wan JJ, Khaw L, et al. Continuous muscle tissue oxygenation in critically injured patients: a prospective observational study. J Trauma. 2006 Oct;61(4):780–8; discussion 788-790.

17. Yu M, Morita SY, Daniel SR, Chapital A, Waxman K, Severino R. Transcutaneous pressure of oxygen: a noninvasive and early detector of peripheral shock and outcome. Shock. 2006 Nov;26(5):450–6.

18. Ahlquist RP. Present state of alpha- and beta-adrenergic drugs I. The adrenergic receptor. Am Heart J. 1976 Nov;92(5):661–4.

19. Intensive Care Society. Levels of Adult Critical Care Second Edition Consensus Statement [Internet]. Intensive Care Society; 2021 [cited 2022 Mar 13]. Available from: https://www.cc3n.org.uk/uploads/9/8/4/2/98425184/2021-03__levels_of_care_second_edition.pdf
