# Supplementary material for: Imaging the choroidal microvasculature in intensive and high dependency care unit patients: a pilot study
Source: BMJ Open. 2026 Feb 25;16(2):e109656. doi: 10.1136/bmjopen-2025-109656 (PMC12958972; doi:10.1136/bmjopen-2025-109656)
Supplement: online supplemental file 2 [file bmjopen-16-2-s002.pdf]

Supplementary Material 2: OCT Imaging Protocol

Optical coherence tomography B-scans were collected using the spectral domain Heidelberg SPECTRALIS Flex module (Heidelberg, Germany) of the right eye. Patients were positioned in either supine, reclined, or erect upright (seated) positions, depending on comfort and with consideration for bedspace ergonomics (Figure 1). Where anterior chamber depth was satisfactory, topical tropicamide was applied to induce mydriasis. All scans collected were macular-centred with a maximum field of view of approximately 9mm. Enhanced depth imaging was activated in order to improve visualisation of the choroidal vessels. Active eye tracking and automatic real time (ART) was used to help improve image quality, longitudinal registration and reduce speckle noise. Our imaging protocol is enumerated below in order of image capture:

1. A fovea-centred, posterior pole horizontal-lined B-scan with an ART of 100
2. A fovea-centred, posterior pole vertical-line B-scan with an ART of 100.
3. A fovea-centred posterior pole volume scan, consisting of equally spaced B-scans, approximately 240 microns apart using an ART of 9. Dependent upon patient cooperation, the field of view for this volume scan was reduced:
    a. Full cooperation: 31 B-scans covering an 8.0 x 6.6 mm region of interest;
    b. Adequate cooperation: 25 B-scans covering an 8.0 x 5.3 mm region of interest;
    c. Challenging cooperation: 25 B-scans a 5.3 x 5.3 mm region of interest.
4. If the patient was still fully cooperative, a circular peripapillary B-scan centred on the optic nerve head using an ART of 100.

Each B-scan comprised a transverse and axial pixel resolution of 768 and 496, respectively. The axial physical resolution was 3.87 microns per pixel. Transverse resolution was variable, ranging from 10-13 microns per pixel (11.68 in emmetropia) with a typical resolution of 496 (axial) x768 (transverse) pixels. The scanning speed was 85kHz.
