# Supplementary material for: Imaging the choroidal microvasculature in intensive and high dependency care unit patients: a pilot study
Source: BMJ Open. 2026 Feb 25;16(2):e109656. doi: 10.1136/bmjopen-2025-109656 (PMC12958972; doi:10.1136/bmjopen-2025-109656)
Supplement: online supplemental file 3 [file bmjopen-16-2-s003.pdf]

Supplementary Material 3: Variables Collected

Variable Name	Units	Measure	Source	Baseline	Follow-Up
Age	Years	Independent	CRF	Yes	No
Sex (Male/Female)	Binary	Independent	CRF	Yes	No
Height	cm	Independent	CRF	Yes	No
Weight	kg	Independent	CRF	Yes	No
BMI	N/A	Independent	CRF	Yes	No
APACHE2	N/A	Independent	CRF	Yes	No
Setting (ITU/HDU)	Binary	Independent	CRF	Yes	No
Diagnosis	Free	Independent	CRF	Yes	No
CCI	N/A	Independent	CRF	Yes	No
SARS-CoV-2 Status	Binary	Independent	CRF	Yes	No
Previous Ophthalmology	Free	Independent	CRF	Yes	No
Additional Comorbidities	Free	Independent	CRF	Yes	No
Hospital Admission	Days	Independent	CRF	Yes	Yes
ITU Admission	Days	Independent	CRF	Yes	Yes
Eye Imaged	Chiral	Process	CRF	Yes	Yes
Flashlight Test Performed	Binary	Process	CRF	Yes	Yes
Flashlight Test Result	Binary	Process	CRF	Yes	Yes
Tropicamide Applied	Binary	Process	CRF	Yes	Yes
Patient Position	Free	Process	CRF	Yes	Yes
Image Time	D-T	Process	CRF	Yes	Yes
Saline (Tears) Applied	Binary	Process	CRF	Yes	Yes
Image Adverse Events	Free	Process	CRF	Yes	Yes
Imaging Challenges	Free	Process	CRF	Yes	Yes
Patient Airway	Free	Independent	CRF	Yes	Yes
Respiratory Therapy	Free	Independent	CRF	Yes	Yes
Fraction Inspired Oxygen	%	Independent	CRF	Yes	Yes
PEEP	cmH_2O	Independent	CRF	Yes	Yes
Vasopressor Required	Binary	Independent	CRF	Yes	Yes
Invasive Blood Pressure	mmHg	Independent	CRF	Yes	Yes
VAECMO Required	Binary	Independent	CRF	Yes	Yes
RRT Required	Binary	Independent	CRF	Yes	Yes
Sedation Infusion	mcg/hr	Independent	CRF	Yes	Yes
Vasopressor Infusion	mcg/hr	Independent	CRF	Yes	Yes
Analgesia Infusion	mcg/hr	Independent	CRF	Yes	Yes
24 Hour Fluid Input	ml	Independent	CRF	Yes	Yes
24 Hour Fluid Output	ml	Independent	CRF	Yes	Yes
24 Hour Fluid Balance	ml	Independent	CRF	Yes	Yes
Cumulative Fluid Input	ml	Independent	CRF	Yes	Yes
Cumulative Fluid Output	ml	Independent	CRF	Yes	Yes
Cumulative Fluid Balance	ml	Independent	CRF	Yes	Yes
Peripheral Pulse Oximetry	%	Independent	CRF	Yes	Yes
Respiratory Rate	bpm	Independent	CRF	Yes	Yes

Systolic Blood Pressure	mmHg	Independent	CRF	Yes	Yes
Diastolic Blood Pressure	mmHg	Independent	CRF	Yes	Yes
Peripheral Capillary Refill Time	seconds	Independent	CRF	Yes	Yes
Glasgow Coma Score	N/A	Independent	CRF	Yes	Yes
Peripheral Temperature	Celsius	Independent	CRF	Yes	Yes
Right Heart Catheterisation	cmH_2O	Independent	CRF	Yes	Yes
Haemocrit	%	Independent	CRF	Yes	Yes
Haemoglobin	g/L	Independent	CRF	Yes	Yes
Albumin	g/L	Independent	CRF	Yes	Yes
Creatinine	mmol/L	Independent	CRF	Yes	Yes
Highest Lactate (24 Hour)	mmol/L	Independent	CRF	Yes	Yes
Highest C-Reactive Protein (24 Hour)	mmol/L	Independent	CRF	Yes	Yes
Choroidal Thickness (Subfoveal)	um	Dependent	OCT	Yes	Yes
Choroidal Area	mm^2	Dependent	OCT	Yes	Yes
Chroroidal Stromal Area	mm^2	Dependent	OCT	Yes	Yes
Choroidal Luminal Area	mm^2	Dependent	OCT	Yes	Yes
Choroidal Vascular Index	N/A	Dependent	OCT	Yes	Yes
Suprachoroid Present	Binary	Dependent	OCT	Yes	Yes
Suprachoroid Thickness	um	Dependent	OCT	Yes	Yes
Image Quality	N/A	Dependent	OCT	Yes	Yes
Complications of Critical Care Admission Between Images	N/A	Process	CRF	No	Yes

CRF: Case Report Form; BMI: Body Mass Index; APACHE2: Acute Physiological and Chronic Health Evaluation; ITU: Intensive Treatment Unit; HDU: High Dependency Unity; CCI: Charleston Comorbidity Index; N/A: Not Applicable; D-T Date-Time; PEEP: Positive End Expiratory Ventilation; VAECMO: Veno-Arterial Extracorporeal Membrane Oxygenation; RRT: Renal Replacement Therapy; OCT: Optical Coherence Tomography.
