# Supplementary material for: Imaging the choroidal microvasculature in intensive and high dependency care unit patients: a pilot study
Source: BMJ Open. 2026 Feb 25;16(2):e109656. doi: 10.1136/bmjopen-2025-109656 (PMC12958972; doi:10.1136/bmjopen-2025-109656)

Supplementary Material 4: Choroid Measurements

Figure 1: Diagram of choroidal measurements of interest for this study.

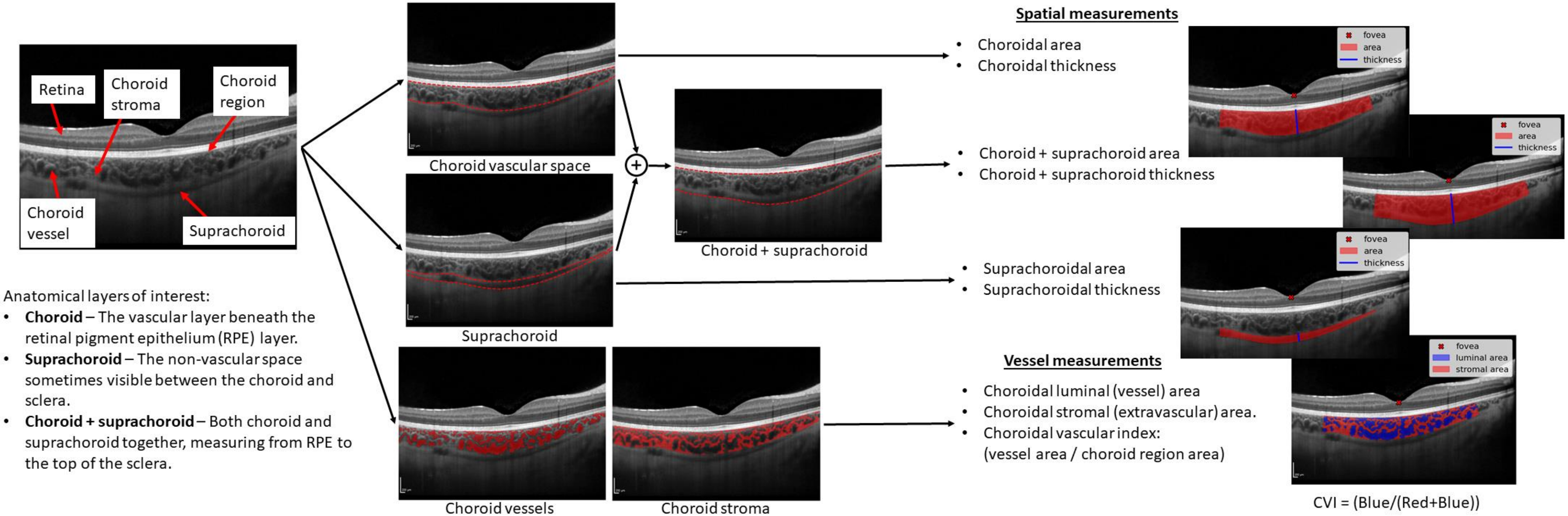

Supplement: online supplemental file 4 [file bmjopen-16-2-s004.pdf]
