# Supplementary material for: Imaging the choroidal microvasculature in intensive and high dependency care unit patients: a pilot study
Source: BMJ Open. 2026 Feb 25;16(2):e109656. doi: 10.1136/bmjopen-2025-109656 (PMC12958972; doi:10.1136/bmjopen-2025-109656)
Supplement: online supplemental file 5 [file bmjopen-16-2-s005.pdf]

Supplementary Material 5: Post-Hoc Analyses.

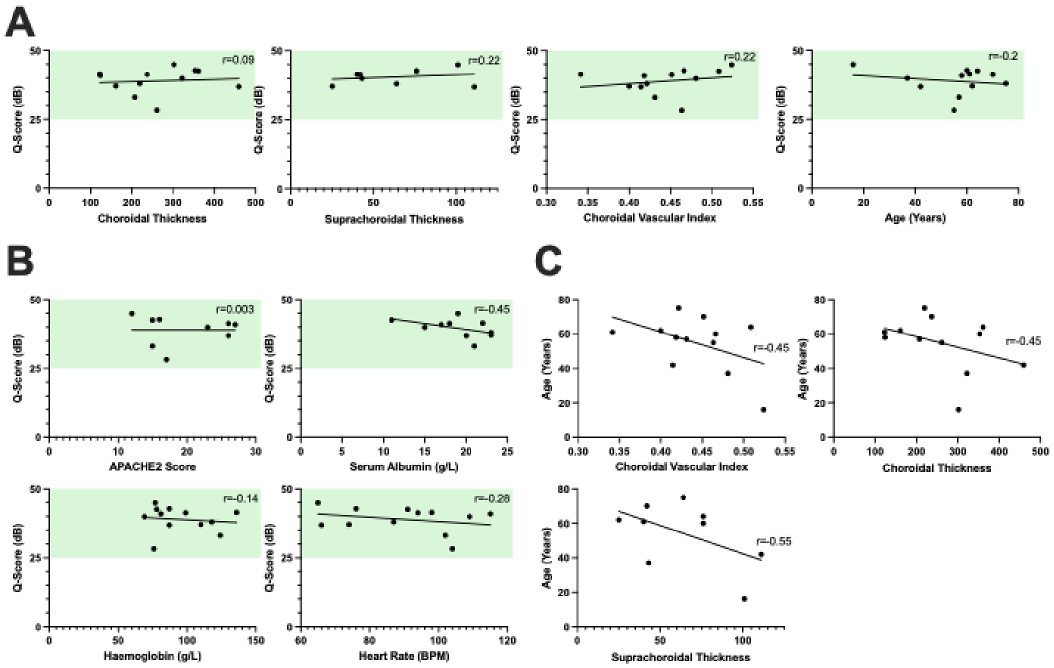

Figure 1 (Above): Sensitivity analyses of (A) Correlation between Heidelberg Q-Score and subfoveal choroidal thickness (n=12), suprachoroidal thickness (n=9), choroidal vascular index (n=12) and age (n=12). (B) Correlation between Heidelberg Q-Score and APACHE2 Score (n=9), serum albumin (g/L, n=10), haemoglobin (g/L, n=12), and heart rate (BPM, n=12). (C) age and subfoveal choroidal vascular index (n=12), choroidal thickness (n=12), and suprachoroidal thickness (n=12). All correlation coefficients are reported and none were statistically significant.

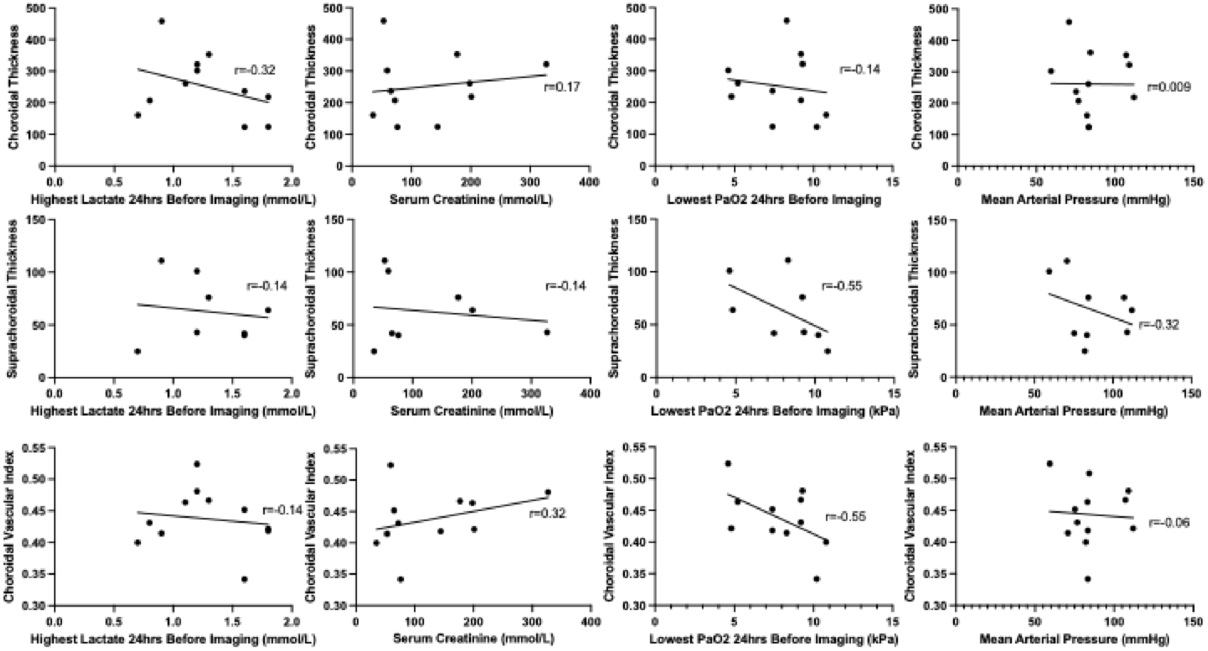

Figure 2 (Above): Post-hoc analyses of subfoveal choroidal thickness, suprachoroidal thickness, and choroidal vascular index against highest lactate 24-hours before imaging (mmol/L), serum creatinine (mmol/L), lowest PaO2 24hrs before imaging (kPa), and mean arterial pressure (mmHg). Missing data points have been excluded (n=8-12).
