# Supplementary material for: Imaging the choroidal microvasculature in intensive and high dependency care unit patients: a pilot study
Source: BMJ Open. 2026 Feb 25;16(2):e109656. doi: 10.1136/bmjopen-2025-109656 (PMC12958972; doi:10.1136/bmjopen-2025-109656)
Supplement: online supplemental file 6 [file bmjopen-16-2-s006.pdf]

Supplementary Material 6: Co-Variation of Intra-Ocular Structures with Fluid Status.

Figure 1: Co-Variation of Cumulative and 24-Hour Fluid Balance with Choroidal Thickness.

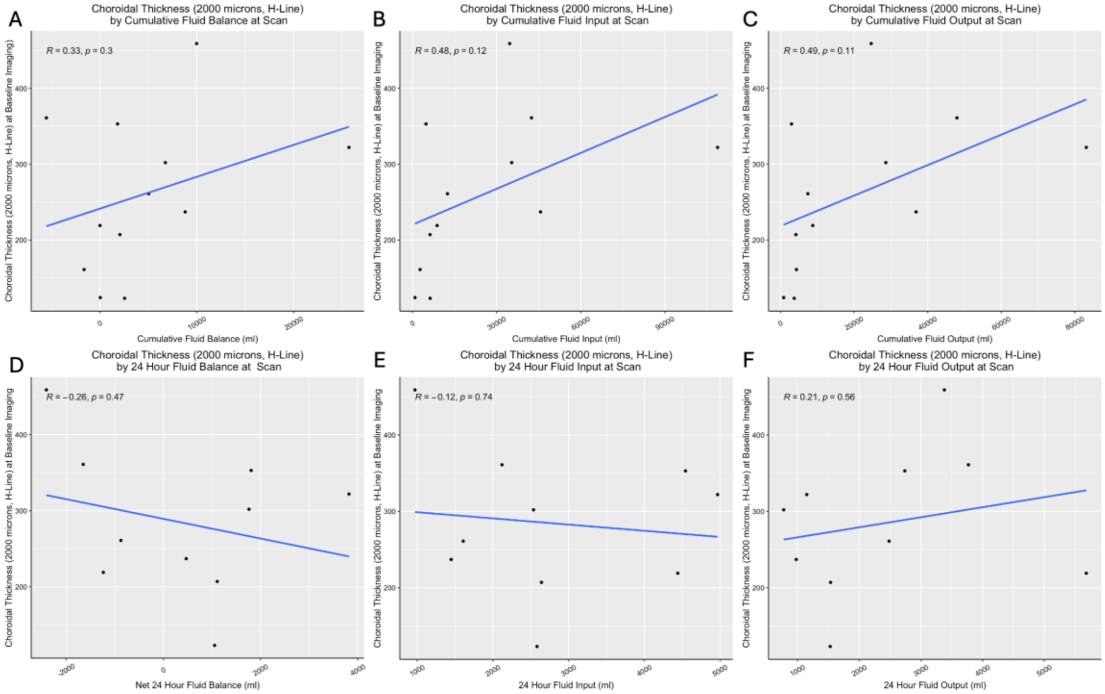

Figure 1: *Univariate analysis of subfoveal choroidal thickness against: A: cumulative fluid balance; B: cumulative fluid input; C: cumulative fluid output; D: 24-hour fluid balance; E: 24-hour fluid input; F: 24-hour fluid output at baseline imaging.*

Figure 2: Co-Variation of Cumulative and 24-Hour Fluid Balance with Suprachoroidal Thickness.

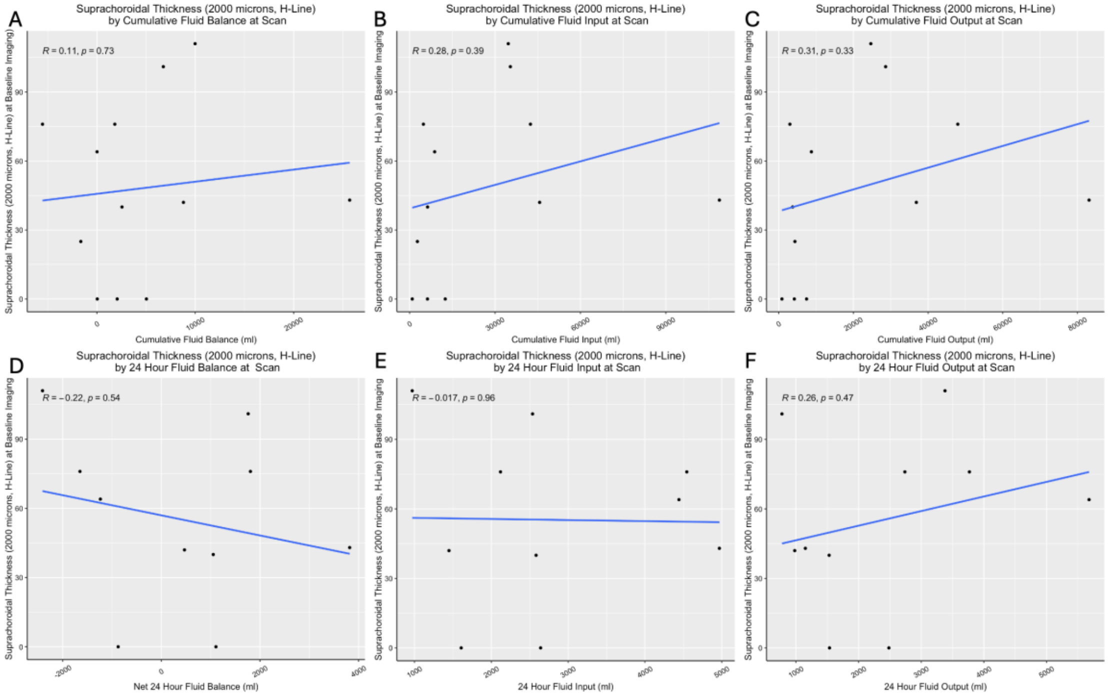

Figure 2: *Univariate analysis of subfoveal suprachoroidal thickness against: A: cumulative fluid balance; B: cumulative fluid input; C: cumulative fluid output; D: 24-hour fluid balance; E: 24-hour fluid input; F: 24-hour fluid output at baseline imaging.*

Figure 3: Co-Variation of Cumulative and 24-Hour Fluid Balance with Total Choroidal Area.

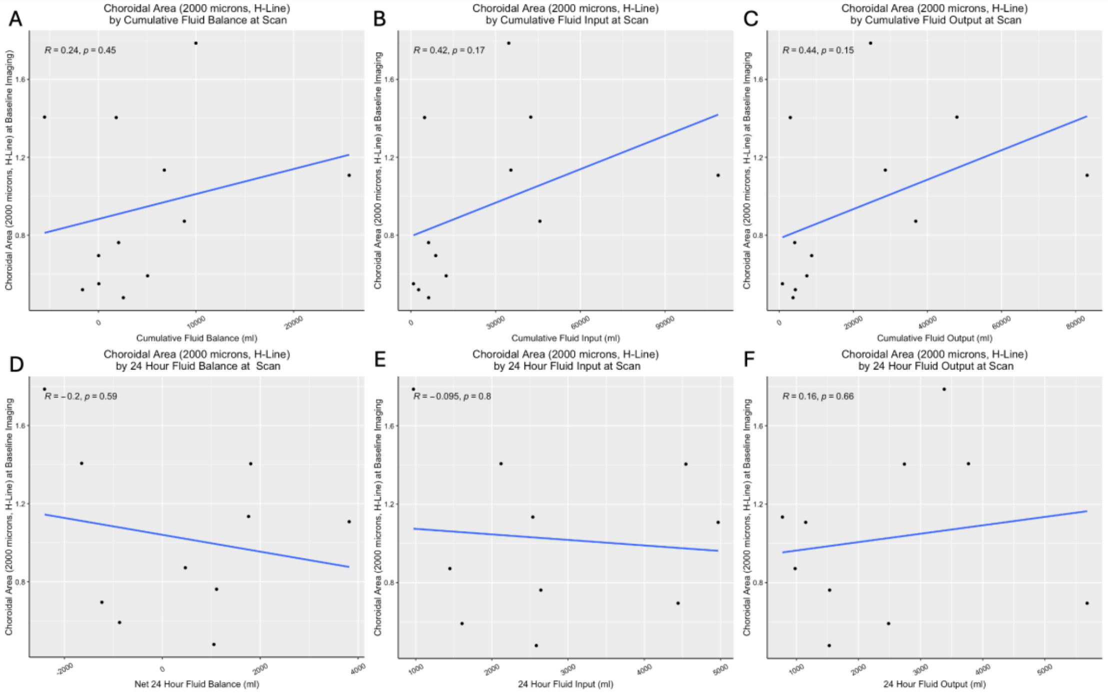

Figure 3: *Univariate analysis of choroidal area against: A: cumulative fluid balance; B: cumulative fluid input; C: cumulative fluid output; D: 24-hour fluid balance; E: 24-hour fluid input; F: 24-hour fluid output at baseline imaging.*

Figure 4: Co-Variation of Cumulative and 24-Hour Fluid Balance with Choroidal Stromal Area.

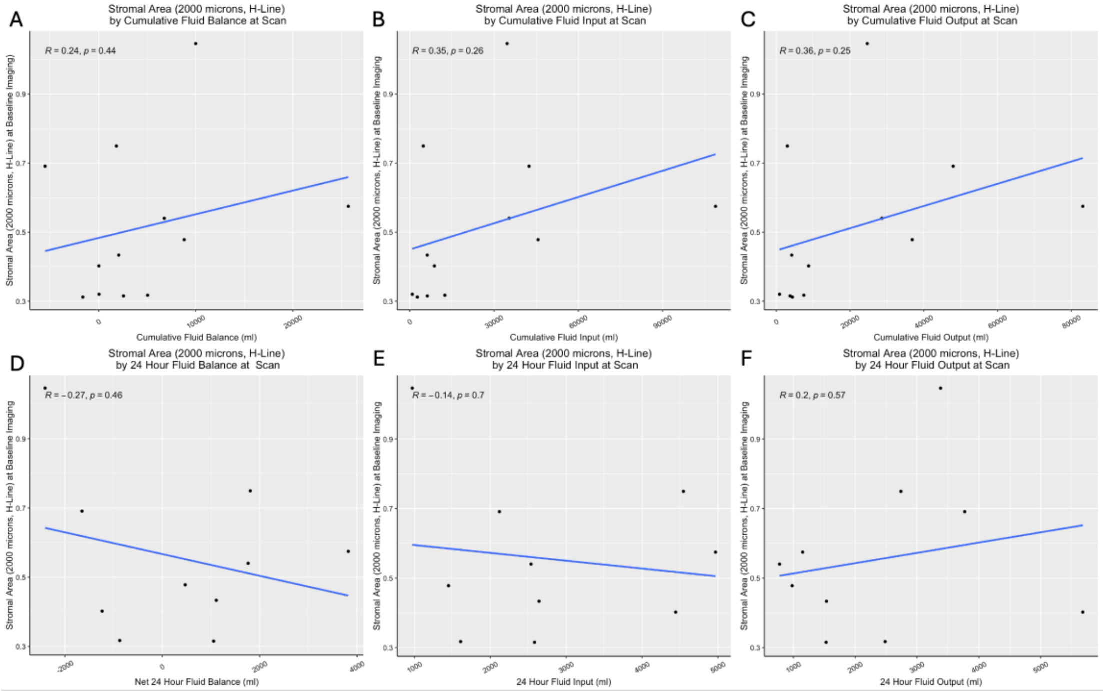

Figure 4: *Univariate analysis of choroidal stromal area against: A: cumulative fluid balance; B: cumulative fluid input; C: cumulative fluid output; D: 24-hour fluid balance; E: 24-hour fluid input; F: 24-hour fluid output at baseline imaging.*

Figure 5: Co-Variation of Cumulative and 24-Hour Fluid Balance with Luminal Area.

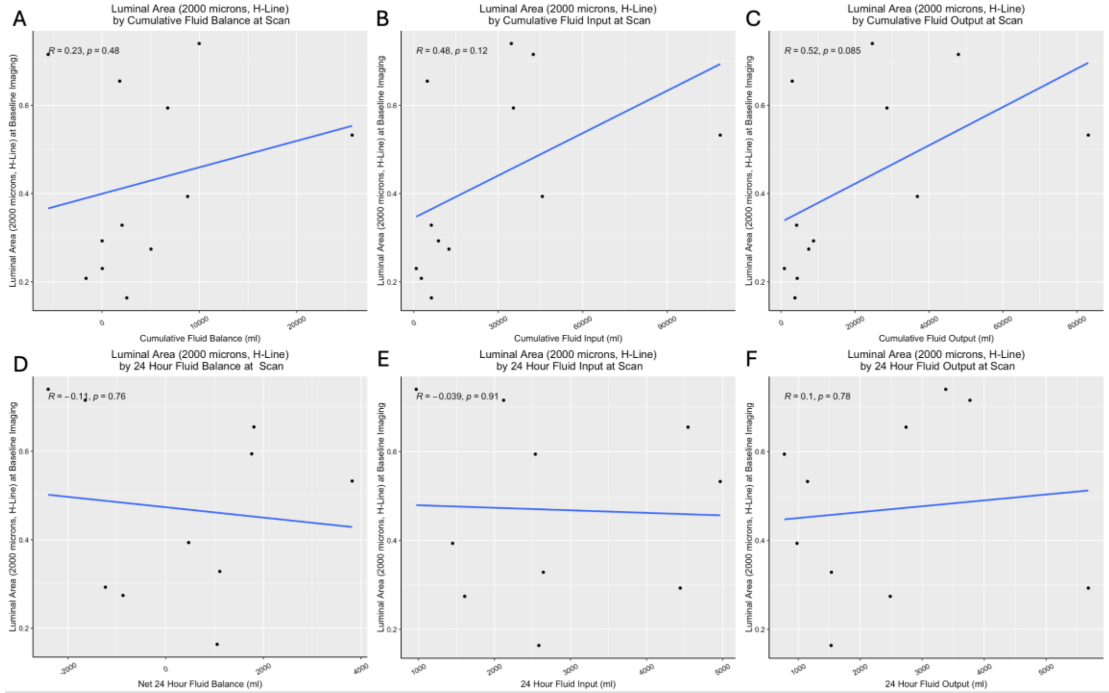

Figure 5: *Univariate analysis of choroidal vascular luminal area against: A: cumulative fluid balance; B: cumulative fluid input; C: cumulative fluid output; D: 24-hour fluid balance; E: 24-hour fluid input; F: 24-hour fluid output at baseline imaging.*

Figure 6: Co-Variation of Cumulative and 24-Hour Fluid Balance with Choroidal Vascular Index.

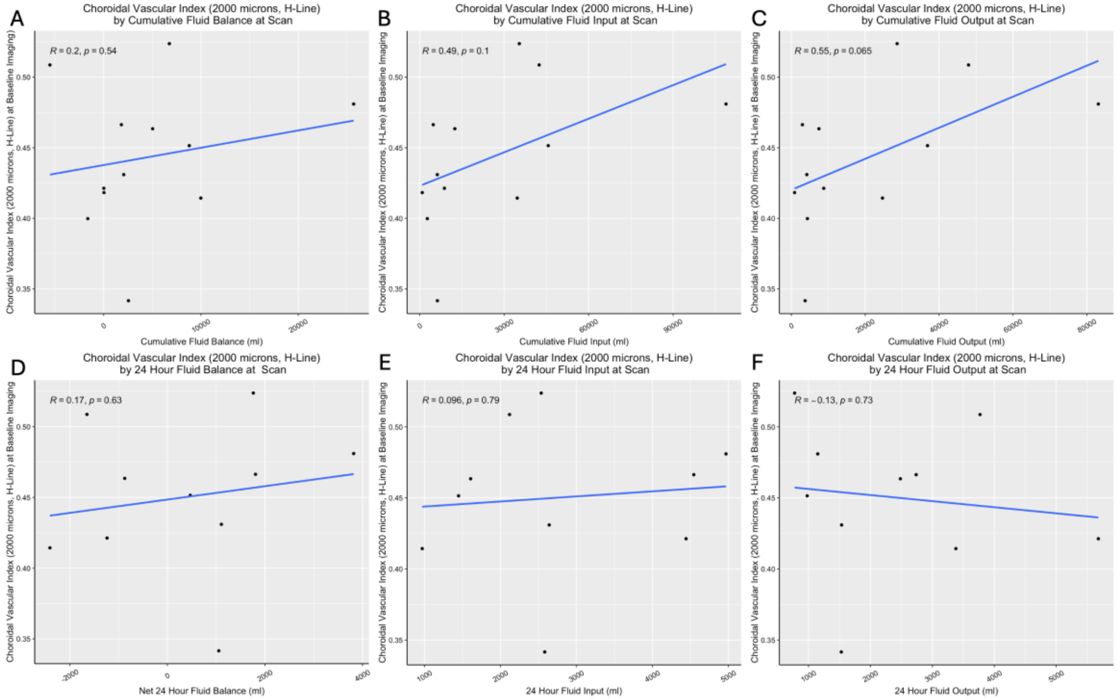

Figure 6: *Univariate analysis of choroidal vascular index against: A: cumulative fluid balance; B: cumulative fluid input; C: cumulative fluid output; D: 24-hour fluid balance; E: 24-hour fluid input; F: 24-hour fluid output at baseline imaging.*
