# Supplementary material for: Imaging the choroidal microvasculature in intensive and high dependency care unit patients: a pilot study
Source: BMJ Open. 2026 Feb 25;16(2):e109656. doi: 10.1136/bmjopen-2025-109656 (PMC12958972; doi:10.1136/bmjopen-2025-109656)
Supplement: Supplementary file 7 [file bmjopen-2025-109656.reviewer_comments.pdf]

PEER REVIEW HISTORY

BMJ Open publishes all reviews undertaken for accepted manuscripts. Reviewers are asked to complete a checklist review form and are provided with free text boxes to elaborate on their assessment. These free text comments are reproduced below.

ARTICLE DETAILS

Title (Provisional)

Imaging the Choroidal Microvasculature in Intensive and High Dependency Care Unit Patients– A Pilot Study

Authors

Cooper, George Max; Burke, Jamie; Hamid, Charlene; Godden, Emily; Dhaun, Neeraj; King, Stuart; MacGillivray, Tom; Baillie, Kenneth; Griffith, David M; MacCormick, Ian JC

VERSION 1 - REVIEW

**Reviewer**	**1**
**Name**	**Chua, Jacqueline**
**Affiliation**	**Singapore National Eye Centre, Singapore Eye Research Institute**
**Date**	**25-Sep-2025**
**COI**	**None**

This is a feasibility study addressing whether enhanced-depth imaging OCT (EDI-OCT) of the choroid can be performed at the ICU bedside and whether choroidal indices correlate with systemic physiology.

Major comments

1 Choroidal thickness and vascular indices show substantial diurnal variation (PMID: 22167095); without time-of-day standardisation, measurements may be noisy and confound associations, particularly in a small cohort. Please include this aspect in the limitations. Alternative approaches such as choriocapillaris flow voids measured from the OCTA may be an option (PMID: 32647298). Please discuss this.

2a Choroidal measurements are strongly dependent on axial length and refractive status (PMID: 35703547). The study did not measure or adjust for axial length, which limits interpretation because differences in ocular biometry could confound associations between choroidal thickness/CVI and systemic physiology. Please discuss this as a limitation.

2b Please define how were quality of scans assessed. Did the 14 subjects have good quality scans? It is likely that the sicker individuals have poorer scan quality such as lower Q score and hence lower choroidal metrics. Please also examine the correlation between Q score and systemic values. Similarly, please correlate the choroidal metrics with age given age impacts choroidal metrics (PMID: 26632821).

3 Figure 3 appears to show only ~9 points for the CVI–APACHE II and other systemic correlations, yet 14 baseline patients were imaged. Please clarify how many eyes/patients contributed to each scatterplot and why some are missing (e.g., poor image quality or missing systemic data).

4 The clinical value proposition is not well defined. Blood tests (e.g., lactate, albumin, haemoglobin, fluid balance markers) are already routinely used to gauge perfusion and vascular leak. The authors should clarify what additional or earlier information choroidal OCT provides. It is difficult to judge how the choroidal metrics would influence decision-making compared with standard laboratory monitoring. This is particularly important given the logistical challenges of performing OCT in critically ill patients, where the added benefit over existing, simple, and rapid blood tests is not obvious.

Minor comments

5 The study appears to only scan and analysis the right eye of patients. Did they choose the right eye of patient #5 since both eyes were scanned?

6 It would strengthen the manuscript to include representative EDI-OCT images illustrating the choroidal and suprachoroidal measurements (e.g., an example of suprachoroidal thickening vs thinning). This would help readers visualise the feasibility claims and the biological signal behind the quantitative indices. For instance, showing a typical image from a patient with high APACHE II and low CVI versus a patient with lower APACHE II and higher CVI would make the quantitative plots more tangible.

**Reviewer**	**2**
**Name**	**Rozec, Bertrand**
**Affiliation**	**University hospital of Nantes**
**Date**	**26-Oct-2025**
**COI**	**None**

In this study, the authors evaluate the feasibility/value of using optical coherence tomography (OCT) to assess microcirculation in critically ill patients.

This is an exploratory, pilot study that is particularly interesting but, in my opinion, has too many limitations to be publishable in its current form.

Major comments:

To test the feasibility of such a method, here is what one would expect:

-Admittedly, the population is a critical care population with a corresponding APACHE II score, but a more homogeneous population would have been preferable (post-operative cardiac surgery or major surgery, sepsis, etc.), with or without vasopressors on admission.

-While the measurement technique is relatively well described, basic logistical elements are missing: ergonomics, installation time. Although the authors are quite clear about the number of people required and the acquisition time, a procedural diagram would be useful, or even a few photographs of the device in a real-life situation. Integration into a patient flow, discomfort and risks to the patient should be better described and evaluated.

-Regarding measurement: although the acquisition time is noted, there is no mention of measurement stability over repeated acquisitions or inter-observer variability (clearly at least two people carried out the acquisition).

-No other method is used; it would have been relevant to compare with another method of measuring microcirculation, such as SDF microscopy or NIRS.

The correlation with elements of haemodynamic management should have been better thought out: none of the ‘classic’ haemodynamic assessment values are used for a possible correlation with the values obtained with the new method (arterial pressure – systolic, diastolic or mean –, lactates, oxygenation, CO_2 gap). The only element of potential interest, in my opinion, is the follow-up of patients over time with fluid balance.

Rather than following a few patients over time, it would have been very relevant to evaluate the impact of vascular filling, for example, on the measurement, or a change in vasopressor infusion for instances!

Minor comments:

-The flowchart, summary and text (method and results) are not always consistent and should be reviewed: how many patients were tested, 12 or 14 out of the 15 recruited?

-The tables and figures are sometimes incorrectly named in the text: e.g. in the feasibility paragraph, line 23, you refer to Table 2 and Figure 3B, but shouldn't it be Figure 2b?

-Sometimes the authors use the abbreviation ITU... what does this correspond to? (ICU ?)

-The description of the population is particularly confusing or inconsistent: why so many patients with BPCIA (6/14!) and so few with cardiovascular pathologies or vasopressor use?

In conclusion, with such a limited number of patients, it would undoubtedly have been preferable to limit the study to a more homogeneous population and focus on what we wanted to measure: one haemodynamic parameter among others!

**Reviewer**	**3**
**Name**	**Kase , Satoru**

**Affiliation**	**Hokkaido University**
**Date**	**30-Oct-2025**
**COI**	**None**

The authors should also show that there was no changes in retinal thickness, that should emphasize their hypothesis.

**Reviewer**	**4**
**Name**	**OKONKWO, OGUGUA**
**Affiliation**	**Eye Foundation Hospital, Lagos, Ophthalmology**
**Date**	**04-Nov-2025**
**COI**	**None**

Dear Authors, please consider the comments below

Title: The title reads better as “Choroidal microvasculature imaging in critically ill….”

Abstract: Please provide a detailed description of the nature of critical illness of patients in the ICU. At what level of illness (e.g., Glasgow Coma Scale) can the patients be described to be at?

What is the indication for measuring the choroidal vascularization and what was measured?

OCTA, not OCT, is more beneficial for assessing choroidal circulation and vasculature. Why was OCT used?

Conclusion: There is no such word as “retinal choroid”. The level of critical illness needs to be included in this paper’s conclusion.

Article:

In the objective of this paper, patients in circulatory shock are mentioned. Was this research specific to this category of patients?

In some sections of the paper, the authors confuse the choroid with the retina. The two should not be used interchangeably, as they are distinct structures, as noted in the introduction.

If the authors only measure choroidal and suprachoroidal thickness, can this be taken as imaging of choroidal microvasculature? In today's ocular imaging, choroidal microvasculature refers to the vasculature of the choroid's different layers, which is better observed using OCT Angiography.

The author should provide details on the technology used to assess the choroidal microvasculature, cube size, including scanning speed, and the axial and transverse

resolutions of the scans, segmentation, etc. If this were handheld, it should be stated. What position was the patient in during the imaging?

The reproducibility of the procedure needs to be thoroughly assessed.

Who acquired the scans? What training in OCT acquisition did this person receive, and for how long? We need to understand the person's experience.

What was acquired should be clearly stated. Was this a thickness scan of the choroid or what else?

How was the acquired image processed?

Describe in detail the technique or method of image acquisition; this should be standardized for all the participants.

Was this a handheld or a tabletop device? If it is not handheld, how can a critically ill patient endure the stress of prolonged sitting? The patient's state of arousal or consciousness should be documented or reported.

What patient preparation was required prior to image acquisition?

Patients with poor fixation cannot have an OCT/OCTA image acquired. Were there patients who were unable to fixate?

What are the disadvantages of the procedure or challenges experienced in imaging critically ill patients? This should be clearly stated and discussed.

Given the patient's condition, will a critically ill patient be able to provide adequate informed consent?

VERSION 1 - AUTHOR RESPONSE

Reviewer 1 comments:

C6: Choroidal thickness and vascular indices show substantial diurnal variation (PMID: 22167095); without time-of-day standardisation, measurements may be noisy and confound associations, particularly in a small cohort. Please include this aspect in the limitations.
R6: Thank you for pointing out the possibility of diurnal variation in choroidal thickness contributing to measurement error in our sample. We note that the time of day does influence choroidal thickness (by an average of 30-40 microns in PMID: 22167095). However, the range of choroidal thickness in our sample was much greater than this (about 130 to 450 microns, fig 2A). Therefore, it seems plausible that diurnal variation in choroidal thickness is likely to contribute relatively little to correlations we observed with choroidal metrics. Nevertheless, we agree that future studies would be improved by taking time of measurements into account and mention this in our discussion:

"…"interpretation of choroidal measurements must also be balanced against both the diverse physiological challenges of ICU admission (including organ dysfunction), the

possibility of variation in subfoveal choroidal thickness with time of day, and with refractive error. Future studies would be strengthened by taking time of choroidal measurements into account, as well as refractive error."

C7: Alternative approaches such as choriocapillaris flow voids measured from the OCTA may be an option (PMID: 32647298). Please discuss this.
R7: OCTA is an option for retinal imaging in ICU patients, but reported success rate is relatively low (~56% for macular OCTA) (PMID: 31699727) – possibly because of the relatively long time needed to acquire good quality OCTA images compared to single-line OCT images (10-20 seconds vs 1-2 seconds). We intentionally used image modalities with short acquisition times because we thought these were likely to be feasible in ICU. We explain this, and mention the potential for OCTA, in the discussion:

"Although we have focused on using OCT to measure the choroid, it is possible to assess choroidal vascularity with OCTA, and this could provide information that is superior or complementary to OCT, if patients are able to comply with the longer acquisition time of OCTA compared to OCT."

C8: Choroidal measurements are strongly dependent on axial length and refractive status (PMID: 35703547). The study did not measure or adjust for axial length, which limits interpretation because differences in ocular biometry could confound associations between choroidal thickness/CVI and systemic physiology. Please discuss this as a limitation.
R8: Thank you for highlighting the association between choroidal thickness and refractive error. We agree that refractive error could contribute to variation in choroidal metrics within our sample. However, we think it is implausible that it would confound associations between choroidal measurements and endpoints, because refractive error is unlikely to be causally related to our dependent variables. A confounder must be causally related to exposure and outcome but not mediate the effect of exposure on outcome (PMID: 23236300). We suggest that the associations we report, which are preliminary and exploratory, were observed despite influences on the choroid from factors such as time of day or refractive error. We have added text to the discussion:

"interpretation of choroidal measurements must also be balanced against both the diverse physiological challenges of ICU admission (including organ dysfunction), the possibility of variation in subfoveal choroidal thickness with time of day, and with refractive error. Future studies would be strengthened by taking time of choroidal measurements into account, as well as refractive error"

C9: Please define how were quality of scans assessed. Did the 14 subjects have good quality scans? It is likely that the sicker individuals have poorer scan quality such as lower Q score and hence lower choroidal metrics. Please also examine the correlation between Q score and systemic values. Similarly, please correlate the choroidal metrics with age given age impacts choroidal metrics (PMID: 26632821).

R9: We used the Q-score to measure scan quality. We have included an analysis of Q-score within Supplement 5 and discussed this within the Results section. There were no statistically significant correlations between Q-score and either age, choroidal or physiological indices.

C10: Figure 3 appears to show only ~9 points for the CVI–APACHE II and other systemic correlations, yet 14 baseline patients were imaged. Please clarify how many eyes/patients contributed to each scatterplot and why some are missing (e.g., poor image quality or missing systemic data).
R10: We have clarified the methods to explain that analysis was conducted on a complete case basis. Imaging was attempted on 14 patients at the first time point. Twelve had satisfactory baseline images for segmentation analysis. Further datapoints were excluded because of absent independent variables, for example: APACHE2 score was not calculated in three patients and could not be determined retrospectively. Similarly, two further patients were excluded due to missing serum albumin. We have amended labelling throughout the manuscript to make it clear how many data were included within each analysis.

C11: The clinical value proposition is not well defined. Blood tests (e.g., lactate, albumin, haemoglobin, fluid balance markers) are already routinely used to gauge perfusion and vascular leak. The authors should clarify what additional or earlier information choroidal OCT provides. It is difficult to judge how the choroidal metrics would influence decision-making compared with standard laboratory monitoring. This is particularly important given the logistical challenges of performing OCT in critically ill patients, where the added benefit over existing, simple, and rapid blood tests is not obvious.
R11: As outlined in the Introduction, macrovascular and systemic markers of perfusion have limited applicability to the microvascular circulatory system, for example, aggressive fluid resuscitation through an early goal directed therapy approach did not influence mortality but increased healthcare costs (PMID: 28320242). Similarly, in subsets of patients it may actually cause harm (PMID: 21615299). Effective assessment of microcirculatory function could help us to better understand, identify, and ultimately treat circulatory shock; however, current techniques to assess the microcirculation are either overly invasive or, through peripheral sampling, do not reflect the physiology of vital organs, making this an exciting potential application for OCT technologies (PMID: 37278760). We have highlighted this in our discussion.

Minor comments

C12: The study appears to only scan and analysis the right eye of patients. Did they choose the right eye of patient #5 since both eyes were scanned?
R12: Bilateral imaging was avoided in this study due to the requirement for mydriatic agents used to optimise image capture and the right eye was chosen for consistency. Patient 5 did not require topical mydriasis due to spontaneous pupil dilatation. Both eyes were imaged to assess procedural ergonomics in alternate chirality; however, the right eye was used in analysis to maintain comparison.

C13: It would strengthen the manuscript to include representative EDI-OCT images illustrating the choroidal and suprachoroidal measurements (e.g., an example of suprachoroidal thickening vs thinning). This would help readers visualise the feasibility claims and the biological signal behind the quantitative indices. For instance, showing a typical image from a patient with high APACHE II and low CVI versus a patient with lower APACHE II and higher CVI would make the quantitative plots more tangible.
R13: Thank you for the suggestion. We note that in Figure 1 we show the difference between initial and follow-up scan in a single patient. While the change in choroidal vascular index is subtle, this does highlight the change in suprachoroidal space.

Reviewer 2 Comments:

C14: To test the feasibility of such a method, here is what one would expect: Admittedly, the population is a critical care population with a corresponding APACHE II score, but a more homogeneous population would have been preferable (post-operative cardiac surgery or major surgery, sepsis, etc.), with or without vasopressors on admission.
R14: Thank you for this suggestion. We chose to keep eligibility criteria broad to help us understand as much as we could about feasibility in the full range of patients treated in a general intensive care unit. We have emphasised this limitation and look forward to developing this in future work.

C15: While the measurement technique is relatively well described, basic logistical elements are missing: ergonomics, installation time. Although the authors are quite clear about the number of people required and the acquisition time, a procedural diagram would be useful, or even a few photographs of the device in a real-life situation. Integration into a patient flow, discomfort and risks to the patient should be better described and evaluated.
R15: We now refer readers to an image of the device in Supplement 2, where we also report our imaging protocol. Our aim with this pilot study was to assess feasibility and explore associations with clinically relevant variables. We agree that assessment of how well imaging can be integrated into existing care pathways would be an important part of future research, should our results be reproduced in independent samples of patients.

C16: Regarding measurement: although the acquisition time is noted, there is no mention of measurement stability over repeated acquisitions or inter-observer variability (clearly at least two people carried out the acquisition).
R16: The aims of our study were to assess the feasibility of imaging the choroid in this patient group and also explore associations between measurements of the choroid and systemic variables. We agree that further work is needed to develop any imaging variable into a practical biomarker – including standardisation of image acquisition, and estimation of reproducibility and repeatability within and across clinical sites. These additional objectives will be incorporated into future work.

C17: No other method is used; it would have been relevant to compare with another method of measuring microcirculation, such as SDF microscopy or NIRS.
R17: Whilst comparison to other available technologies will be important future developments this work was outside the scope of the current work.

C18: The correlation with elements of haemodynamic management should have been better thought out: none of the 'classic' haemodynamic assessment values are used for a possible correlation with the values obtained with the new method (arterial pressure – systolic, diastolic or mean –, lactates, oxygenation, CO2 gap). The only element of potential interest, in my opinion, is the follow-up of patients over time with fluid balance.
Rather than following a few patients over time, it would have been very relevant to evaluate the impact of vascular filling, for example, on the measurement, or a change in vasopressor infusion for instances!

R18: The mean time from ICU admission to baseline was 9.1 days. Consequently, many patients had been acutely stabilised prior to baseline imaging, for example, the mean highest lactate 24hrs prior to baseline imaging was 1.54±1.02 (reference 0.5-1.6); similarly, mean arterial pressure was 84.0±15.3 (Table 1). Creatinine concentration and lowest PaO2 in the 24hrs preceding imaging were also collected and are now reported within Table 1 as well. Due to sample size and normalcy, these analyses are underpowered and we did not wish to introduce multiplicity to our analysis. Post-hoc exploratory analysis exploring relationships with OCT indices has now been added to Methods and reported within the appendix as scatter plots. Other suggested indices (e.g. PaCO2/PvCO2 data, change in vasopressor infusions) were not measured but will be considered in future work, thank you.

Reviewer 2 minor comments:

C19: The flowchart, summary and text (method and results) are not always consistent and should be reviewed: how many patients were tested, 12 or 14 out of the 15 recruited?
R19: We agree that due to missing data for some variables, it is not always clear how many patients are included in each analysis. We have improved the language within the Results section and the Figure 3 legend to make this more apparent.

C20: The tables and figures are sometimes incorrectly named in the text: e.g. in the feasibility paragraph, line 23, you refer to Table 2 and Figure 3B, but shouldn't it be Figure 2b?
R20: Figures and table references have been checked throughout and amended appropriately.

C21: Sometimes the authors use the abbreviation ITU... what does this correspond to? (ICU ?)

R21: Apologies, ITU is sometimes used interchangeably with ICU. We have corrected this.

C22: The description of the population is particularly confusing or inconsistent: why so many patients with BPCIA (6/14!) and so few with cardiovascular pathologies or vasopressor use?
R22: We assume the reviewer refers to table 1 "Intra-aortic balloon pump: 6/14 patients". Thank you for pointing out this inconsistency which prompted a review of the source data for this field for the whole cohort. During the review, we found that there were no patients in the analysis cohort had a balloon pump and on questioning research nurses, the use of an arterial line (sometimes called intra-arterial blood pressure (IABP) measurement) had been confused with the use of an intra-aortic balloon pump (also sometimes denoted IABP). We have corrected this error in Table 1.

It should be noted that the mean time to imaging was 9.1±8.8 days and therefore many patients had been stabilised and weaned from vasopressors at the time of this feasibility study. We have mentioned this within the limitations section.

C23: In conclusion, with such a limited number of patients, it would undoubtedly have been preferable to limit the study to a more homogeneous population and focus on what we wanted to measure: one haemodynamic parameter among others!
R23: Thank you for your thorough review of our manuscript. We appreciate your comments.

<u>Reviewer 3 comments</u>

C24: The authors should also show that there was no changes in retinal thickness, that should emphasize their hypothesis.
R24: Retinal thickness was not quantified as our aim was to assess the thickness of the choroid. Choroidal thickness is known to vary independently of retinal thickness in healthy people (PMID: 22167095)

<u>Reviewer 4 comments:</u>

C25: Title: The title reads better as "Choroidal microvasculature imaging in critically ill…."
R25: Thank you for the suggestion. We agree and have amended the title to reflect this.

C26: Abstract: Please provide a detailed description of the nature of critical illness of patients in the ICU. At what level of illness (e.g., Glasgow Coma Scale) can the patients be described to be at?
R26: We have amended the abstract in response to your comments. The Acute Physiology and Chronic Health Evaluation (APACHE2) score is routinely used to stratify critical illness on ICU admission and has now been reported.

C27: What is the indication for measuring the choroidal vascularization and what was measured?

R27: We have further clarified our intra-ocular outcome measures. We are interested in whether microcirculatory dysfunction in critical illness is associated with intra-ocular structural changes. To do this, we focussed our investigation within the choroidal and adjacent structures, primarily choroidal thickness, suprachoroidal space thickness, and the ‘choroidal vascular index’: the proportional volume of the choroid occupied by blood vessels.

C28: OCTA, not OCT, is more beneficial for assessing choroidal circulation and vasculature. Why was OCT used?
R28: See R7.

C29: Conclusion: There is no such word as “retinal choroid”. The level of critical illness needs to be included in this paper’s conclusion.
R29: We have clarified this terminology, thank you. The word ‘choroid’ can refer to several structures containing many blood vessels, for example the choroid plexus in the cerebral ventricles, and so we initially specified that our target was the choroid between the retina and sclera. The level of critical illness is reported through APACHE2 score. All patients were in receipt of Intensive Care Society Level 2 (HDU) or Level 3 (ICU) care.

C30: Article: In the objective of this paper, patients in circulatory shock are mentioned. Was this research specific to this category of patients?
R30: Whilst circulatory shock was not an inclusion criterion and indeed, only three patients (21%) of the cohort had a vasopressor requirement and the time of imaging, this pilot study was envisioned to assess the feasibility an ocular biomarker for microcirculatory perfusion in patients with shock. Following this proof of concept, future work will seek to evaluate this technique within a larger population .

C31: In some sections of the paper, the authors confuse the choroid with the retina. The two should not be used interchangeably, as they are distinct structures, as noted in the introduction.
R31: We have amended and clarified this language.

C32: If the authors only measure choroidal and suprachoroidal thickness, can this be taken as imaging of choroidal microvasculature? In today's ocular imaging, choroidal microvasculature refers to the vasculature of the choroid's different layers, which is better observed using OCT Angiography.
R32: Thank you. We have previously justified our reasons for avoiding OCTA in this population (see R7). Choroidal vascular index has been widely measured from OCT and correlated with physiological and pathophysiological states as a microcirculatory biomarker (PMID: 40915427).

C33: The author should provide details on the technology used to assess the choroidal microvasculature, cube size, including scanning speed, and the axial and transverse resolutions of the scans, segmentation, etc. If this were handheld, it should be stated. What position was the patient in during the imaging?

R33: Patient position and scanning time are presented in Table 2, and we have included the former within the Results section. We were unable to include an image of the Heidelberg Spectralis Flex due to figure restrictions; however, this is a boom-mounted OCT device (see: PMID: 31699727). We have clarified this within the Methods section and included a reference where pictures are demonstrated.

The axial physical resolution was 3.87 microns per pixel. Transverse resolution was variable, ranging from 10-13 microns per pixel (11.68 in emmetropia) with a typical resolution of 496 (axial) x768 (transverse) pixels. The scanning speed was 85kHz, which has been amended within Supplement 2.

Further scan dimensions and segmentation distance (240 microns) are included within Supplementary Material 2. Choroidal microvasculature was assessed using the methodologies outlined here:

- Engelmann J, Burke J, Hamid C, Reid-Schachter M, Pugh D, Dhaun N, et al. Choroidalyzer: An open-source, end-to-end pipeline for choroidal analysis in optical coherence tomography [Internet]. arXiv; 2023 [cited 2024 Mar 15]. Available from: http://arxiv.org/abs/2312.02956
- Burke J. Choroidal image analysis for OCT image sequences with applications in systemic health [Internet]. 2025 [cited 2025 Aug 14]. Available from: http://arxiv.org/abs/2502.07117

C34: The reproducibility of the procedure needs to be thoroughly assessed.

R34: We agree that translation of choroidal imaging into a clinical biomarker would require assessment of repeatability and reproducibility, both within a single centre and across multiple centres. However, our aim in this pilot study was limited to assessing feasibility and exploring associations with clinically relevant dependent variables. We suggest that future work incorporate tests of reproducibility into their protocols.

C35: Who acquired the scans? What training in OCT acquisition did this person receive, and for how long? We need to understand the person's experience.
R35: OCT was performed by trained clinical imagists who have experience utilising this technique and equipment. This information is now included in the Methods.

C36: What was acquired should be clearly stated. Was this a thickness scan of the choroid or what else?
R36: Supplement 4 outlines the full list of parameters collected from OCT imaging. Within each OCT section, these were: choroidal thickness, suprachoroidal thickness, total choroidal area, choroidal vascular area, choroidal stromal area, and suprachoroidal area. The choroidal vascular index is a volumetric proportion of the choroidal vascular area compared with the total choroidal area. To provide an anatomically consistent and clinically replicable comparison, the subfoveal choroidal and suprachoroidal spaces were compared.

C37: How was the acquired image processed?

R37: The segmentation technique is described in the following methodological papers (cited within the text):

- Burke J, Engelmann J, Hamid C, Reid-Schachter M, Pearson T, Pugh D, et al. An Open-Source Deep Learning Algorithm for Efficient and Fully Automatic Analysis of the Choroid in Optical Coherence Tomography. Translational Vision Science & Technology. 2023 Nov 21;12(11):27.
- Burke J, King S. Edge Tracing Using Gaussian Process Regression. IEEE Transactions on Image Processing. 2022;31:138–48.
- Engelmann J, Burke J, Hamid C, Reid-Schachter M, Pugh D, Dhaun N, et al. Choroidalyzer: An open-source, end-to-end pipeline for choroidal analysis in optical coherence tomography [Internet]. arXiv; 2023 [cited 2024 Mar 15]. Available from: http://arxiv.org/abs/2312.02956
- Burke J. Choroidal image analysis for OCT image sequences with applications in systemic health [Internet]. 2025 [cited 2025 Aug 14]. Available from: http://arxiv.org/abs/2502.07117

C38: Describe in detail the technique or method of image acquisition; this should be standardized for all the participants.
R38: This is outlined in Supplementary 2.

C39: Was this a handheld or a tabletop device? If it is not handheld, how can a critically ill patient endure the stress of prolonged sitting? The patient's state of arousal or consciousness should be documented or reported.
R39: As mentioned, the device is boom mounted (PMID: 31699727). Table 2 summarises patient position (60% supine) and GCS score (mean 13.2). This has been signposted more clearly within the body of the Results section.

C40: What patient preparation was required prior to image acquisition?
R40: As set out in the Methods section and Supplementary 2, patients only received topical mydriasis following reassuring oblique illumination test, prior to imaging.

C41: Patients with poor fixation cannot have an OCT/OCTA image acquired. Were there patients who were unable to fixate?
R41: Active eye tracking and automatic real time technologies are incorporated within the Heidelburg Flex, facilitating imaging in patients with poor fixation. As set out in Supplement 2 (Imaging Protocol), the field of view was adjusted in response to patient cooperation to facilitate capture. Table 2 summarises challenges associated with imaging on a case-by-case basis.

C42: What are the disadvantages of the procedure or challenges experienced in imaging critically ill patients? This should be clearly stated and discussed.
R42: Procedural challenges are presented in Table 2 and signposted within the updated Methods and Results sections.

C43: Given the patient's condition, will a critically ill patient be able to provide adequate informed consent?

C43: Patients without capacity are unable to provide informed consent. Under the Adults with Incapacity Act 2000 (Scotland), legal provision can be made to recruit a patient into a research study. This study was designed in accordance with these principles, for example, the steps taken within the protocol design to minimise material risk and alleviate burden (e.g., patient public involvement). This process is overseen by the Scotland A Research Ethics Committee who issued a favourable opinion on this study (22/SS/0055). This has been emphasised within the Methodology section.

VERSION 2 - REVIEW

Reviewer 1

Name Chua, Jacqueline

Affiliation Singapore National Eye Centre, Singapore Eye Research Institute

Date 22-Jan-2026

COI

Thank you. I have no further comments. I am satisfied with the revision.

Reviewer 2

Name Rozec, Bertrand

Affiliation University hospital of Nantes

Date 31-Jan-2026

COI

The authors have largely answered my questions.

Nevertheless, due to a population that was certainly hospitalized in intensive care but ultimately not very representative of critically ill intensive care patients (notably the absence of shock at the time of the examination), I remain skeptical about the value of these examinations for their care. As for feasibility, I have no doubt that an ophthalmologist is capable of performing this type of examination... but what is the potential in terms of monitoring and follow-up of intensive care patients?

I am not convinced.

Reviewer 4

Name OKONKWO, OGUGUA

Affiliation Eye Foundation Hospital, Lagos, Ophthalmology

Date **02-Feb-2026**

COI

The authors have clarified several of the issues I raised in the first round of review.

However, it is necessary to indicate the area in the manuscript where changes or additions were made.

I suggest that the authors include a photograph of the boom-mounted device in use. The readers will be interested in seeing this.

VERSION 2 - AUTHOR RESPONSE

Reviewer 1:
C2: Thank you. I have no further comments. I am satisfied with the revision.
R2: Thank you very much for your contributions to this paper.

Reviewer 2:
C3: The authors have largely answered my questions.
R3: We are grateful for your insights and the challenges you have highlighted translating this work into clinical practice.

C4: Nevertheless, due to a population that was certainly hospitalized in intensive care but ultimately not very representative of critically ill intensive care patients (notably the absence of shock at the time of the examination), I remain sceptical about the value of these examinations for their care.
R4: Thank you, we recognise that this is a limitation within this work, as the study design limited point-of-admission testing. Within this revision, we have expanded this within the limitations section:

"Reflecting this constraint and the pragmatic limitations of this study, the mean time from critical care admission to imaging was 9.1 days, which limited the utility and applicability of certain biomarkers (e.g. lactate, vasopressor requirement) following the initial stabilisation of these patients. Future work should aim to image patients closer to or during admission, where the effects of acute physiological disturbances may be more apparent, whilst also accounting for any variation from axial eye length and refractive error, and time of day, all of which can influence choroidal thickness.[22,23]" [Page 12-13].

We have also highlighted this a threat to applicability and a consideration for future work:

"Finally, this heterogeneity limits the interpretation and applicability of our choroidal evaluation to specific diagnoses. Future work will seek to evaluate intraocular structures within more homogenous patient populations." [Page 13-14].

C5: As for feasibility, I have no doubt that an ophthalmologist is capable of performing this type of examination… but what is the potential in terms of monitoring and follow-up of intensive care patients? I am not convinced
R5: Thank you for this comment, we recognise that this will have future implications to the feasibility of this technology in wider clinical practice and have further amended our limitations section to reflect this.
There are several handheld OCTs currently available and in development across industry (https://siloton.com/about/) and academia (https://alumni.liv.ac.uk/news/stories/title,1515342,en.html). We envision that further advancements will improve the imaging depth and ergonomics of these devices, facilitating usage within inpatient and non-ophthalmologic teams and have further highlighted this within our discussion:

"We anticipate that the size, cost, imaging depth, and ease of use of portable and handheld OCT equipment is likely to improve over time.[24] The advent and improvement of handheld OCT devices could facilitate diagnostic ergonomics, helping create a potential role for ocular microcirculatory biomarkers in the management of critically ill patients, throughout both their inpatient admission and follow-up.[25,26] Open-source analytics for retinochoroidal OCT images would further facilitate this, to create a 'closed-loop' device for non-expert users.[14–16] The primary source of attrition was discharge from critical care (50%, n=4/8) and, in addition to incorporating handheld OCT where feasible, future work should include administrative and ethical provisions to administer patients in lower level inpatient care settings.[27]" [Page 13].

We also note that ongoing work evaluating retinopathy of prematurity in Neonatal Intensive Care Unit patients at Duke University (USA), further highlights the feasibility and demonstrates the potential utility of this technology for adult ICU patients (PMID 39808124).

Future work will seek to understand whether variance within intraocular structures persists throughout hospital admission and into outpatient settings within specific disease groups. It is hoped that this will further elucidate microcirculatory adaption to critical illness and facilitate biomarker development for ICU and post-ICU patients.

Reviewer 4:
C6: The authors have clarified several of the issues I raised in the first round of review.
R6: Thank you very much

C7: However, it is necessary to indicate the area in the manuscript where changes or additions were made.
R7: A marked copy of the manuscript will be included within the revision, we hope this satisfactorily highlights where changes and additions have been made. Changes from the first revision are in red, with second revision changes further highlighted in yellow.

C8: I suggest that the authors include a photograph of the boom-mounted device in use. The readers will be interested in seeing this.
R8: Within the methods of this revision (page 7, paragraph 3), we signpost a photo of this device in literature (Figure 1, Liu et al. 2019: PMID: 31699727).
